# Modulation of Supramolecular Interaction of Pt(II) Complexes Bearing Carbene Cyclometalate for Color-Tunable Luminescence

**DOI:** 10.1021/acs.inorgchem.5c04898

**Published:** 2025-12-03

**Authors:** Yixin Wu, Lin Cheng, Yu-Cheng Kung, Yi Pan, Shek-Man Yiu, Jie Yan, Kai Li, Wen-Yi Hung, Yun Chi, Kai-Chung Lau

**Affiliations:** a Department of Chemistry, Department of Materials Science and Engineering, and Center of Super-Diamond and Advanced Films (COSDAF), 53025 City University of Hong Kong, Kowloon 999077, Hong Kong SAR; b Department of Optoelectronics and Materials Technology, 34880 National Taiwan Ocean University, Keelung 20224, Taiwan; c Guangdong Provincial Key Laboratory of New Energy Materials and Service Safety, Shenzhen Key Laboratory of New Information Display and Storage Materials, College of Materials Science and Engineering, 47890 Shenzhen University, Shenzhen 518071, China

## Abstract

Pt­(II) complexes are known for their versatility in showing aggregation-induced emission. Herein, we report four Pt­(II) complexes **Pt-1**, **Pt-2**, and **Pt-3a/b** featuring both the carbene cyclometalate (C^C) and chelating pyrazolate (N^N). We evaluated two synthetic methods: the first employed the carbene intermediate **Pt-DMSO**, followed by N^N coordination to give **Pt-1-Cl** and aryl cyclometalation, while the second involved sequential addition of C^C and N^N chelates to Pt­(COD)­Cl_2_. These Pt­(II) complexes exhibited efficient sky-blue emission in a PMMA matrix at high dilution. Upon increasing the doping ratio, emission of **Pt-1** and **Pt-3a** remained essentially unchanged, but **Pt-2** and **Pt-3b** exhibited gradual emergence of a low-energy band at ∼575 nm, which was attributed to the metal–metal-to-ligand charge transfer emission band. These aggregation propensities aligned well with the time-dependent density functional theory calculations based on the dimers and trimers. For applications, the organic light-emitting diode devices based on **Pt-1** exhibited blue emission with a max. external quantum efficiency (EQE) of 11.7% at 50 wt %, while **Pt-2**-based devices gave concentration-dependent emission with max. EQEs of 9.4, 11.3, and 7.1% and CIE_*xy*_ coordinates of (0.26, 0.45), (0.33, 0.47), and (0.45, 0.49) at 10, 50, and 100 wt %, respectively.

## Graphical abstract

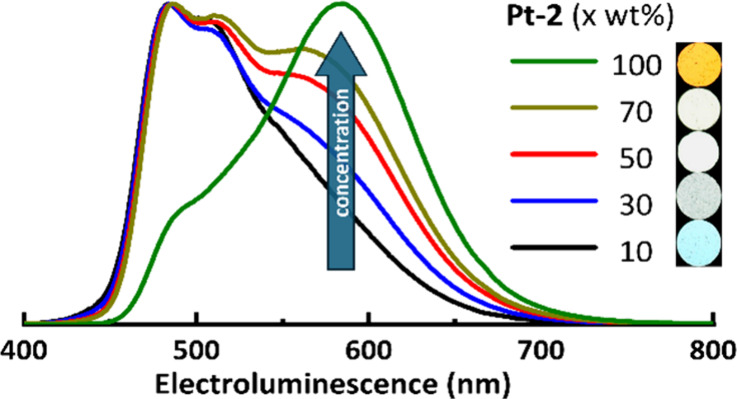

## Introduction

Emissive transition-metal complexes are integral to modern coordination chemistry and have been pivotal to the development of organic light-emitting diodes (OLEDs). The two most studied families of emissive complexes are those with central iridium­(III) and platinum­(II) atoms, to which their high spin–orbit coupling constant facilitated fast singlet-to-triplet intersystem crossing and allowed generation of efficient phosphorescence at room temperature.^1,2^ This property makes relevant Ir­(III) or Pt­(II) complexes the ideal OLED emitters in harvesting otherwise wasted triplet excitons that were generated in a theoretical 3:1 ratio vs singlet excitons during operation. In fact, Ir­(III) emitters are already used in commercial OLED devices in green and red, while Pt­(II) emitters are promising phosphors for future development of robust blue OLED devices.^3–5^

For construction of efficient transition-metal emitters, a wide range of chromophoric chelates have been reported, including N-heterocyclic carbene cyclometalates (C^C),^6^ N-heteroaromatic cyclometalates (C^N),^7^ and pyridinyl pyrazolate (N^N).^8,9^ Scheme 1 gave representative examples of these chelates, namely, 1-methyl-3-phenyl-1*H*-imidazol-2-ylidene (**pmi**),^10,11^ 2-phenylpyridine (**ppy**),^12^ and 2-(1*H*-pyrazol-5-yl)­pyridine (**pzpy**),^13^ and all of them possess one minus formal charge and are capable to coordinate to both Ir­(III) and Pt­(II) metal atoms in giving a diverse range of brightly emissive phosphors. Modification can be also executed to incorporate a plethora of functional groups that could confer color-tuning and exhibit diversified photophysical properties.^14^ Their coordination atom on chelate (i.e., carbon vs nitrogen) strongly influences the crystal field strength of final metal complexes, among which the (C^C) carbene chelates would offer better emission efficiency due to the larger energy gap between the emissive excited state (cf. the T_1_ state) and upper-lying metal-centered (MC) dd quenching state.^15^

**Scheme 1:**
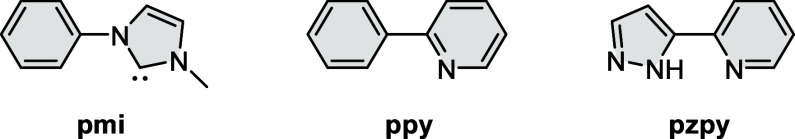
Representative Examples of (C^C), (C^N), and (N^N) Chelates for Forming Transition-Metal Complexes

In general, unlike their Ir­(III) congeners with a maxima of three carbene cyclometalates,^16,17^ there is no report on Pt­(II) metal complexes featuring two bidentate carbene cyclometalates.^18^ Pt­(II) complexes carried only one bidentate carbene cyclometalate, those with ancillary that was derived from neutral donors such as phosphine,^19–21^ pyridine,^22^ nitrile, and isocyanide^23^ and anions such as cyanide,^24,25^ acetylide,^26–28^ acetylacetonate,^29–31^ and others that can be found in the literature. Notably, those with charge-neutral phosphine- and pyridine-based ancillary ligands tended to give cationic Pt­(II) complexes, while those with dual anionic ligands afford anionic Pt­(II) complexes. As expected, the ionic Pt­(II) complexes would suffer from poor volatility and cannot be employed for the fabrication of an OLED using direct vacuum deposition. On the other hand, promising charge-neutral Pt­(II) carbene complexes can be found with those featuring bidentate ancillary such as acetylacetonate,^29–31^ bis­(pyrazolyl)­borate,^32,33^ and formamidinate,^34^ to which their structures are depicted in Scheme 2 for scrutiny. However, only a handful of examples were reported, and the majority displayed inferior OLED performances.

**Scheme 2:**
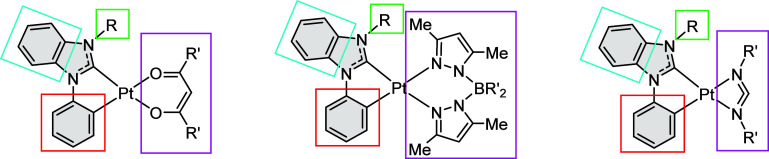
General Motif of Pt­(II) Complexes with Bidentate Carbene and Ancillary, with Colored Boxes Showing Functionalization Sites

In this work, we sought to prepare Pt­(II) complexes featuring both bidentate carbene (C^C) and pyrazolate (N^N) chelates for achieving better emission efficiency and color tunability. After that, synthesis, characterization, and theoretical calculations were performed on two parallel classes of Pt­(II) complexes. Their structure–property relationship allowed an in-depth exploration of factors that affected their emission color vs aggregation behavior in different states and hence OLED performances.

## Results and Discussion

### Synthesis and Characterizations

Execution of this research project required two distinctive carbene pro-chelates **(LAH**_**2**_ **)­(BF**_**4**_ **)** and **(LBH**_**2**_ **)­(OTf)** and two bidentate pyrazolate chelates **(im** ^**i**^ **z)­H** and **(fppz)­H**, to which their structural drawings are depicted in Scheme 3. First, the symmetrical carbene chelate **(LAH**_**2**_ **)­(BF**_**4**_ **)** was made from diamination of 1,2-dibromo-4,5-dimethylbenzene in giving intermediate **A1**, followed by cyclization of **A1** with trimethyl orthoformate in affording the benzo­[*d*]­imidazol-3-ium entity, cf. Scheme S1 of the Supporting Information (SI). The second pro-chelate **(LBH**_**2**_ **)­(OTf)** exhibited an asymmetric imidazo­[4,5-*b*]­pyridin-3-ium fragment and was obtained using 2-chloro-3-nitropyridine as the starting material, following a three-step protocol as shown in Scheme S2. Concurrently, the pyrazolate chelates with distinctive steric encumbrance and intrinsic electronic character, namely, 5-(1-isopropyl-1*H*-imidazol-2-yl)-3-(trifluoromethyl)-1*H*-pyrazole **(im** ^**i**^ **z)­H** and 2-(3-(trifluoromethyl)-1*H*-pyrazol-5-yl)­pyridine **(fppz)­H**, were prepared in accordance with literature methods.^35,36^ The divergent structures of these carbene (C^C) and pyrazolate (N^N) chelates give synthesized Pt­(II) complexes with varied photophysical properties in both fluid and thin-film states.

**Scheme 3:**
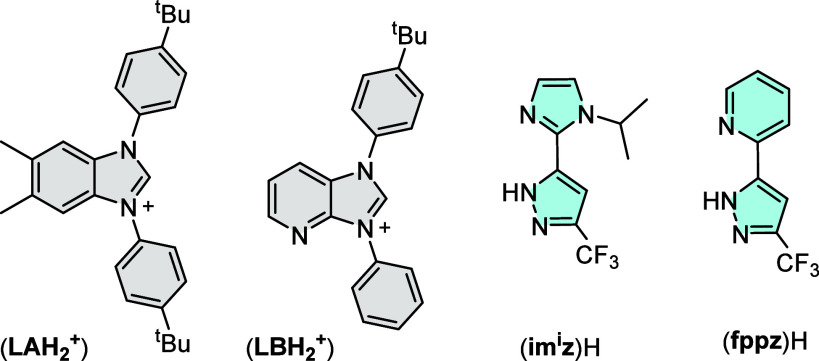
Drawings of Carbene and N-Donor Chelates Employed in the Present Study

Next, we employed the symmetric carbene chelate **(LAH**_**2**_ **)­(BF**_**4**_ **)** to demonstrate the pathways that could afford the emissive Pt­(II) complex featuring one carbene cyclometalate and one bidentate pyrazolate chelate, i.e., **Pt-1**. As shown in Scheme 4, the carbene can be incorporated into the Pt­(II) metal center as an isolable intermediate **Pt-DMSO**. This Pt­(II) complex is structurally analogous to Pt­(II) derivatives with the general formula *cis*-(NHC)­(DMSO)­PtCl_2_ reported in the literature,^37,38^ to which the ^1^H NMR spectrum exhibited a unique signal at δ 2.88 franked with ^195^Pt isotope satellites (*J*_Pt–H_ = 10.2 Hz) that can be assigned to the methyl groups of coordinated DMSO molecules. Next, addition of **(im** ^**i**^ **z)­H** to **Pt-DMSO** resulted in efficient replacement of one chloride and DMSO in giving the second intermediate **Pt-1-Cl**. Its single-crystal X-ray structural data are depicted in Figure S1 of the SI, together with the essential metric parameters. As seen, the molecule exhibited a *trans*-chloride-to-pyrazolate and *trans*-carbene-to-imidazolyl arrangement. The benzo­[*d*]­imidazolylidene also underwent a 90° rotation against the square-planar Pt­(II) framework for avoiding the excessive steric congestion between ligands. Finally, prolonged heating of **Pt-1-Cl** with sodium acetate at 185 °C in *o*-dichlorobenzene for 72 h afforded the anticipated Pt­(II) complex **Pt-1** in essentially quantitative yield. The observed retardation in reactivity is probably caused by the **im** ^**i**^ **z** chelation, which may increase the activation barrier for C–H activation of N-aryl appendages on monodentate carbene.

**Scheme 4:**
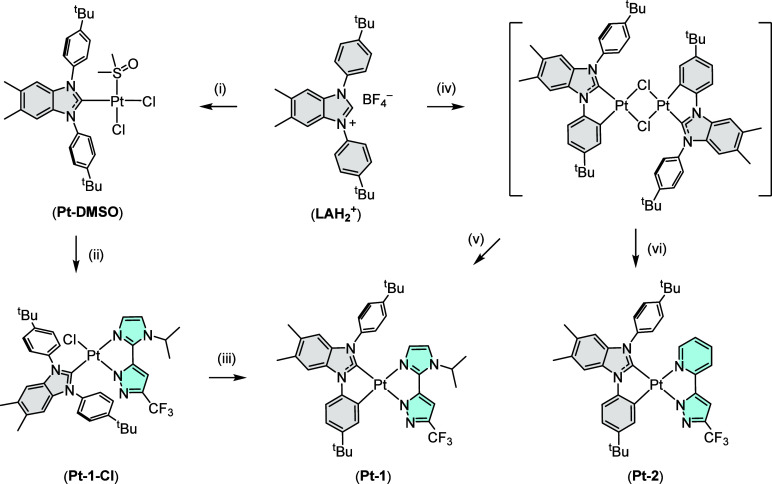
Stepwised Synthesis of Pt­(II) Complexes **Pt-1** and **Pt-2** ^a^

a Experimental conditions: (i) Pt­(DMSO)_2_Cl_2_, NaOAc, acetone/DMSO (v/v = 5/1), reflux; (ii) (im^i^_6_H_4_Cl_2_, reflux; (iv) Pt­(COD)­Cl_2_, NaOAc, DMF, 80 °C; (v) (im^i^z)­H, NaOAc, DMF, 120 °C; (vi) (fppz)­H, NaOAc, DMF, 120 °C.

Alternatively, the same Pt­(II) complex can be obtained using a two-step, one-pot approach. It involves treatment of **(LAH**_**2**_ **)­(BF**_**4**_ **)** with a different metal reagent Pt­(COD)­Cl_2_ and sodium acetate in DMF solution, followed by addition of a second N-donor **(im** ^**i**^ **z)­H**. Due to the absence of coordinative DMSO molecules, aryl cyclometalation is expected to occur concomitantly with carbene coordination in giving a hypothetical dimer intermediate supported by two bridging chloride ligands.^24,39–41^ Characterization of this intermediate was unfortunately hampered by its poor stability during isolation and purification. However, its existence has been confirmed by several related precedents reported in the literature.^28,34^ Even with this ambiguity, formation of **Pt-1** can be achieved in high yield upon addition of **(im** ^**i**^ **z)­H** with a significant reduction in reaction time and temperature, i.e., 4 h at 120 °C using a one-pot approach vs 72 h at 185 °C from **Pt-1-Cl**. The obvious time savings made the second approach a better strategy in giving these Pt­(II) complexes. Hence, the corresponding pyridinyl derivative **Pt-2** was obtained from **(LAH**_**2**_ **)­(BF**_**4**_ **)** and a different pyrazolate chelate **(fppz)­H** using the second method. Similarly, one-pot coordination of **(LBH**_**2**_ **)­(OTf)** and **(im** ^**i**^ **z)­H** gave two distinctive Pt­(II) complexes **Pt-3a** and **Pt-3b**, to which their production is caused by cyclometalation at either the N-aryl site, cf. Scheme 5. Finally, high-temperature equilibration was observed in the presence of both a proton and an acetate catalyst without notable decomposition. This chemical transformation is related to those observed in the Ir­(III) carbene complexes,^42–45^ confirming the high thermodynamic stability of these Pt­(II) metal complexes.

**Scheme 5:**
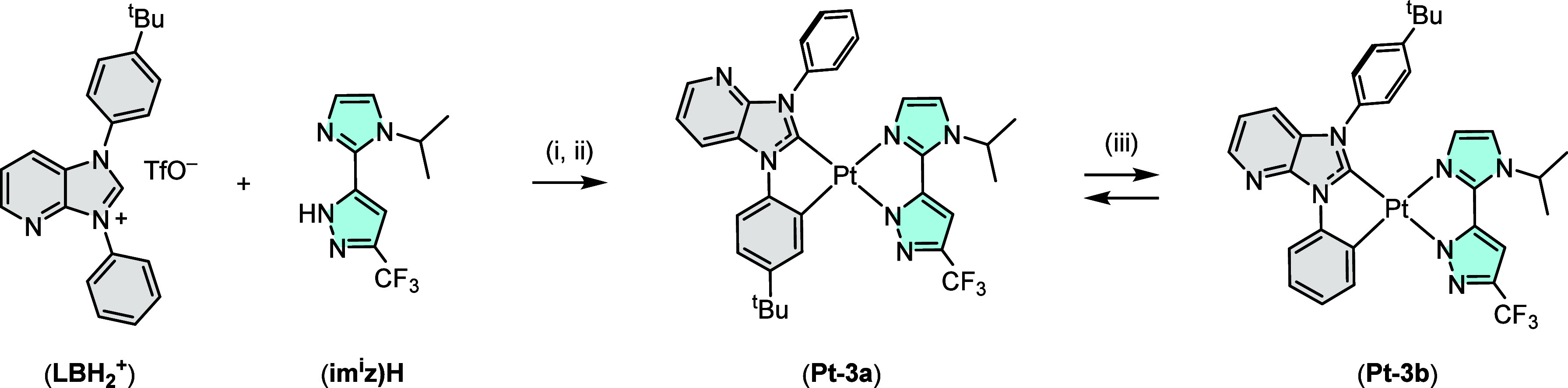
One-Pot Coordination of **(LBH**_**2**_ **)­(OTf)** and **(im** ^**i**^ **z)­H** on Pt­(II) Metal Center^a^

a Experimental conditions: (i) Pt­(COD)­Cl_2_, NaOAc, DMF, 80 °C; (ii) NaOAc, DMF, 120 °C; (iii) TsOH·H_2_O, NaOAc, 214 °C, reflux.

Next, single-crystal X-ray structural analyses on **Pt-1**, **Pt-2**, **Pt-3a**, and **Pt-3b** were executed to decipher their exact structures in the solid state, which are depicted in Figures 1–4, respectively. To all Pt­(II) complexes, the neutral carbene entity and negatively charged aryl cyclometalate are both located at the *trans*-disposition to the pyrazolate and neutral imidazolyl (or pyridinyl) group, to which their structures are reminiscent of Pt­(II) complexes with nominal *cis*-oriented N-aromatic cyclometalates.^46–50^ Moreover, all Pt–C­(carbene) distances are notably shorter than the respective Pt–C­(cyclometalate) bond, while Pt–N­(pyrazolate) distances are also shorter than the respective Pt–N distances of imidazolyl or pyridinyl entities. This variation in Pt–C and Pt–N distances is attributed to the greater π-accepting strength of carbene entities and additional electrostatic attraction between the positively charged Pt­(II) metal cation and negatively charged pyrazolate anions. Rotation of the noncoordinated N-aryl group was also observed, cf. 61.7° (**Pt-1**), 58.3° (**Pt-2**), 63.8° (**Pt-3a**), and 58.6° (**Pt-3b**), to which the torsion angle against the central carbene skeleton turned smaller than that observed in **Pt-1-Cl** (∼90°), which possesses the less bulky *cis*-chloride ligand. Moreover, this interchelate steric interaction also forced these Pt­(II) complexes to adopt a slightly distorted and nonplanar architecture.

**Figure 1:**
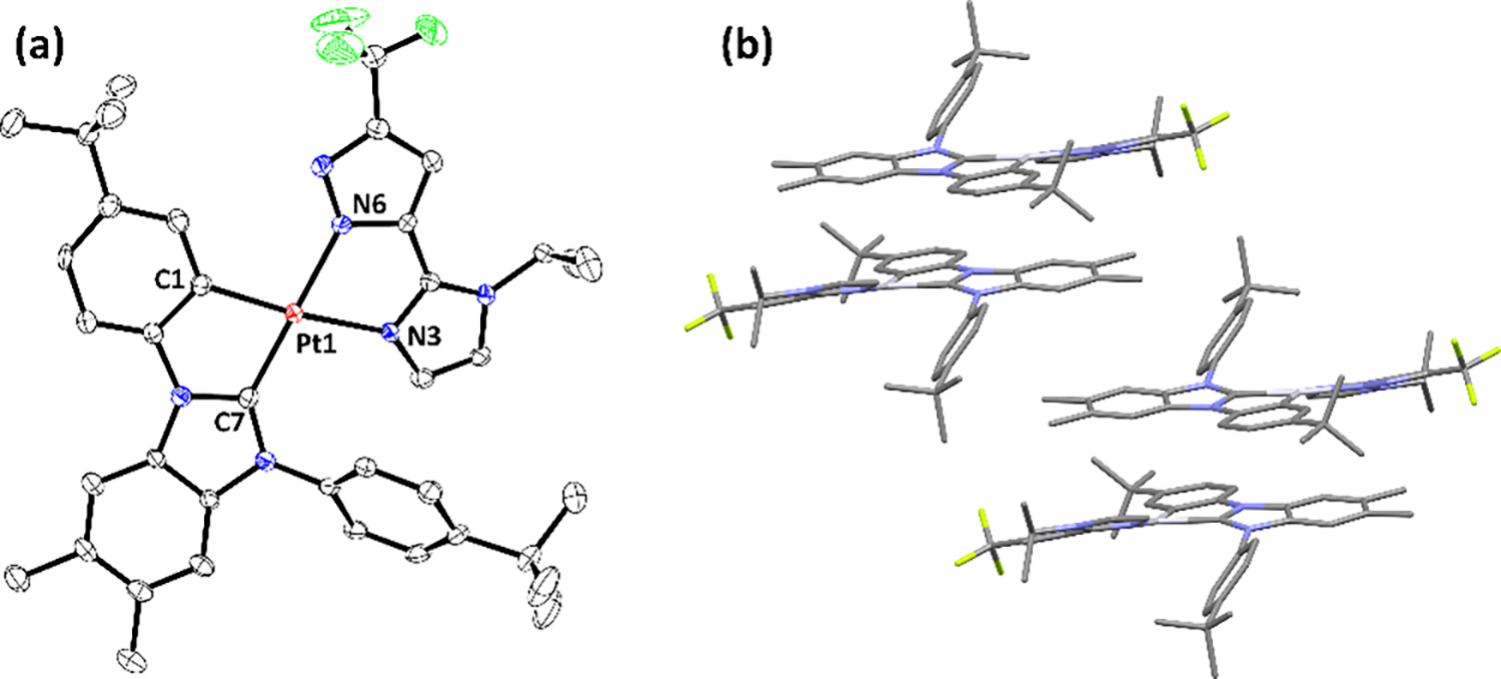
(a) Structure drawing of **Pt-1** with thermal ellipsoids shown at the 30% probability level. Selected bond lengths (Å): Pt1–C(7) = 1.968(6), Pt1–C(1) = 2.006(7), Pt1–N(6) = 2.066(5), and Pt1–N(3) = 2.138(5). Selected bond angles (°): C(7)–Pt1-C(1) = 79.7(3), N(3)–Pt1-N(6) = 76.7(2), C(7)–Pt1-N(6) = 175.8(2), and C(1)–Pt1-N(3) = 170.5(2). Hydrogen atoms were omitted for clarity. (b) Packing diagram of **Pt-1** with the closest Pt···Pt separation of 5.991 Å.

**2:**
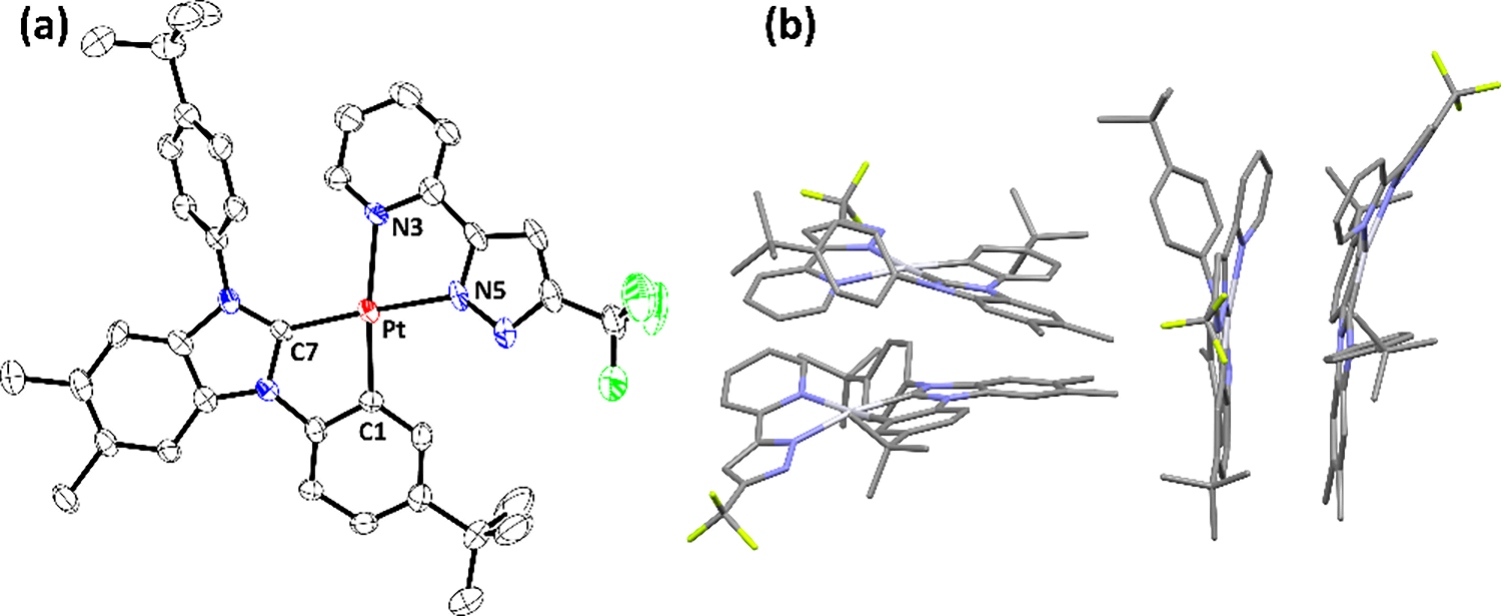
(a) Structural drawing of **Pt-2** with thermal ellipsoids shown at the 30% probability level. Selected bond lengths (Å): Pt1–C(7) = 2.006(9), Pt1–C(1) = 2.022(9), Pt1–N(3) = 2.159(7), and Pt1–N(5) = 2.056(7). Selected bond angles (°): C(7)–Pt1-C(1) = 79.6(4), N(3)–Pt1-N(5) = 77.0(3), C(7)–Pt1-N(5) = 169.0(3), and C(1)–Pt1-N(3) = 168.8(3). Hydrogen atoms were omitted for clarity. (b) Packing diagram of **Pt-2** with the closest Pt···Pt separation of 4.596 Å.

**3:**
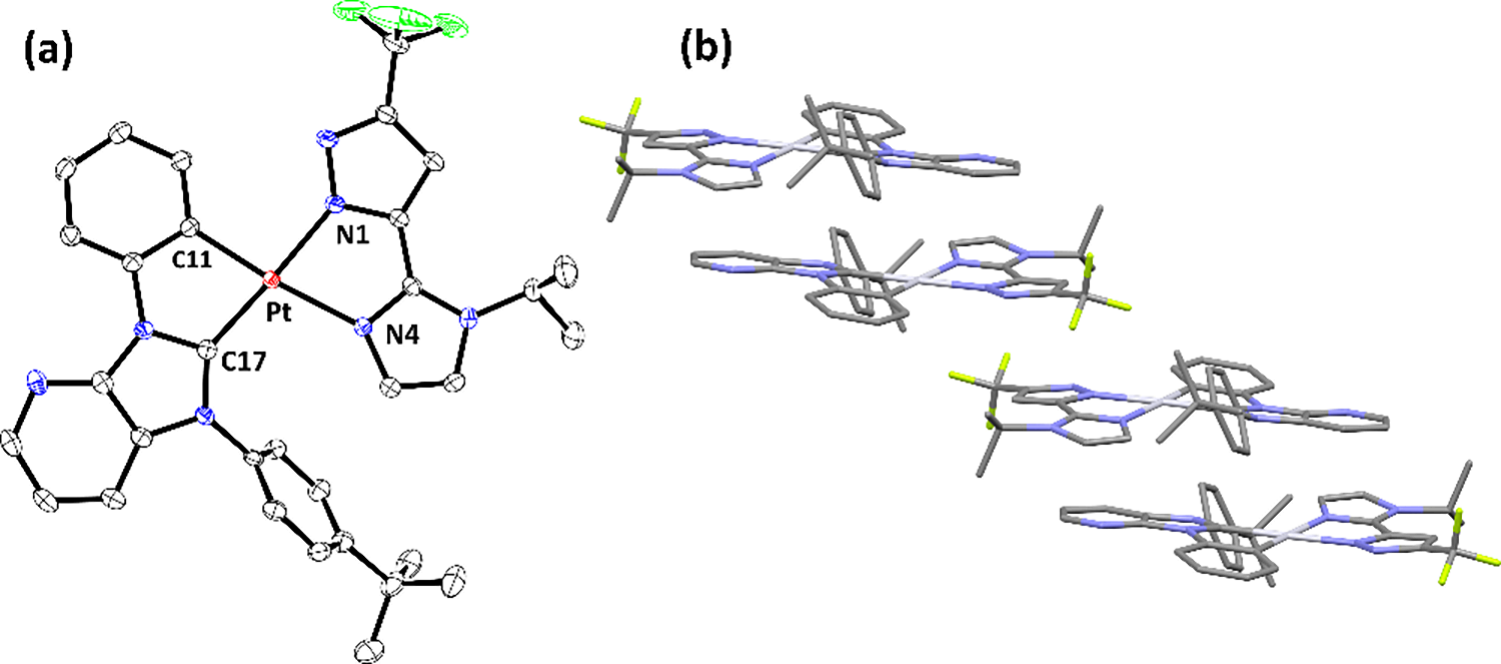
(a) Structure drawing of **Pt-3a** with thermal ellipsoids shown at the 30% probability level. Selected bond lengths (Å): Pt1–C(7) = 1.974(5), Pt1–C(1) = 2.021(5), Pt1–N(1) = 2.059(4), and Pt1–N(4) = 2.149(4). Selected bond angles (°): C(7)–Pt1-C(1) = 79.4(2), N(1)–Pt1-N(4) = 76.82(16), C(7)–Pt1-N(1) = 175.86(18), and C(1)–Pt1-N(4) = 172.46(18). Hydrogen atoms were omitted for clarity. (b) Packing diagram of **Pt-3a** with the closest Pt···Pt separation of 5.107 Å.

**Figure 4:**
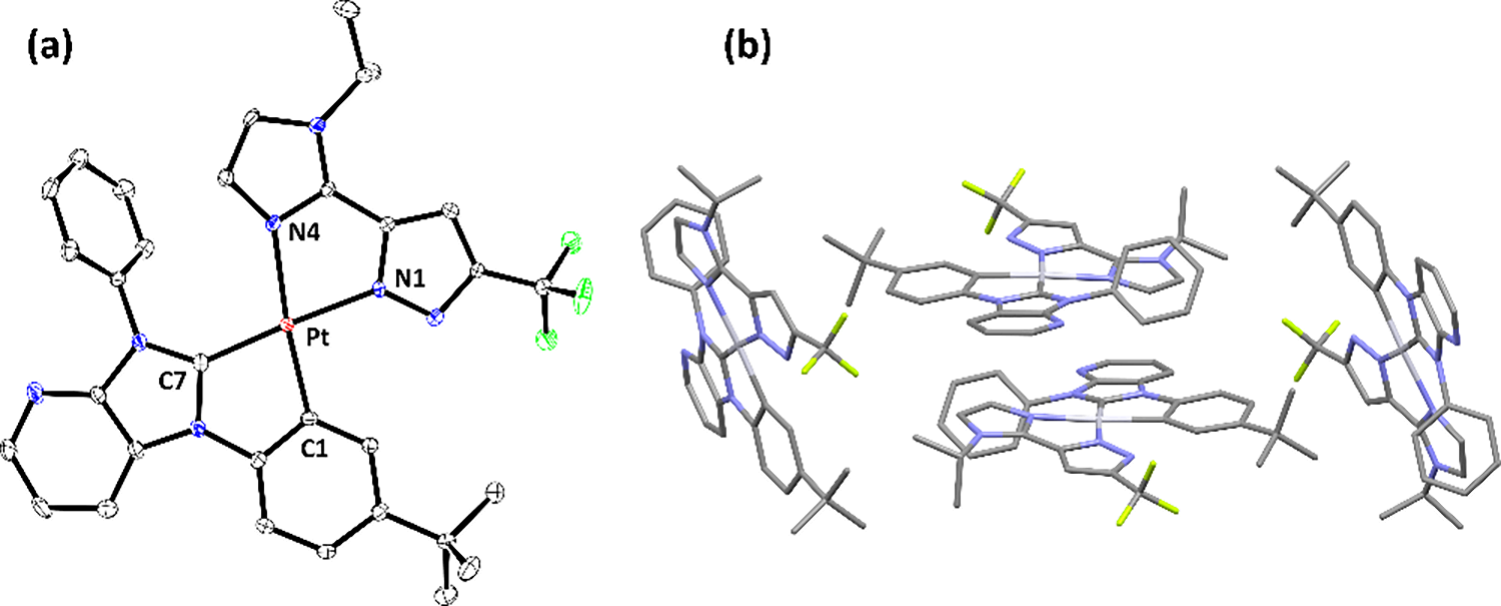
(a) Structure drawing of **Pt-3b** with thermal ellipsoids shown at the 30% probability level. Selected bond lengths (Å): Pt1–C(17) = 1.975(4), Pt1–C(11) = 2.009(4), Pt1–N(1) = 2.063(4), and Pt1–N(4) = 2.141(3). Selected bond angles (°): C(17)–Pt1-C(11) = 79.72(16), N(1)–Pt1-N(4) = 77.10(13), C(17)–Pt1-N(1) = 176.97(15), and C(11)–Pt1-N(4) = 174.29(14). Hydrogen atoms were omitted for clarity. (b) Packing diagram of **Pt-3b** with the closest Pt···Pt separation of 5.197 Å.

For the intermolecular stacking in the crystal lattice, Pt­(II) complexes **Pt-1** and **Pt-2**, both adopting dual *t*-butylphenyl-substituted carbene **LA**, existed as a virtual dimer with the closest Pt···Pt contacts of 5.991 and 4.596 Å, respectively. The shortened Pt···Pt contact for **Pt-2** is obviously caused by the lack of an isopropyl substituent on pyridinyl chelate **fppz** in reference to the imidazolyl chelate **im** ^**i**^ **z** of **Pt-1**. Then, it allowed the adjacent Pt­(II) molecules to better align with each other, giving the reduced Pt···Pt separation. In fact, these packing structures are in accordance with their mechanochromic properties, to which grinding the yellow-colored crystals displayed no obvious change in emission for imidazolyl **Pt-1** but afforded a notable change of emission from light-green to orange for pyridinyl counterpart **Pt-2** instead.

In sharp contrast, the second set of Pt­(II) complexes **Pt-3a** and **Pt-3b**, both possessing the less congested carbene **LB** with only one *t*-butylphenyl group on the imidazo­[4,5-*b*]­pyridin-2-ylidene entity, displayed distinctive dimer-like and slip chain arrangement with comparable Pt···Pt separations of 5.107 and 5.197 Å, respectively. Interestingly, despite having slightly longer nonbonding Pt···Pt contact for **Pt-3b**, its slip chain arrangement may have greater flexibility in achieving better intermolecular packing upon application of external forces. This is confirmed by the change of emission from sky-blue to orange upon grinding the crystals, i.e., mechanochromism that was observed in the literature.^51,52^ We will return to this issue while talking about the photophysical behavior of doped thin films at different concentrations and theoretical investigation (*vide infra*).

### Photophysical, Electrochemical, and Thermal Characterizations

The intermediate **Pt-1-Cl** showed no discernible emission due to the lower-lying MC dd excited state induced by the π-donating chloro ligand, and hence, no attempt was made to discuss its photophysical properties. Alternatively, absorption spectra of Pt­(II) complexes **Pt-1**, **Pt-2**, and **Pt-3a/b** were recorded in toluene with a conc. of 10^–5^ M at RT, to which the graphic and numeric data are depicted in Figure 5 and Table 1. As can be seen, **Pt-2** exhibited a ligand-centered π–π absorption band at 369 nm, which is more redshifted than its **im** ^**i**^ **z** counterparts **Pt-1** and **Pt-3a/b** (347 and 354/355 nm, respectively), attributed to the pyridine-based **fppz** chelate with greater π conjugation. In terms of photoluminescence, Pt­(II) complexes **Pt-1** and **Pt-3a/b** exhibited a structured emission profile bearing two max. at ∼484 and ∼503 nm, which are consistent with the dominant π–π* transition character. This assignment is further confirmed with the large Stokes shift from their lowest-energy π–π* absorption band. In sharp contrast, **Pt-2** exhibited broadened dual emission bands with one peak max. located at ∼490 nm and second at ∼591 nm, which are attributed to the normal π–π* emission as well as the excimer emission. This agreed with the literature reports on Pt­(II) complexes that showed a strong tendency in forming dimers upon excitation.^47,53–63^

**Figure 5:**
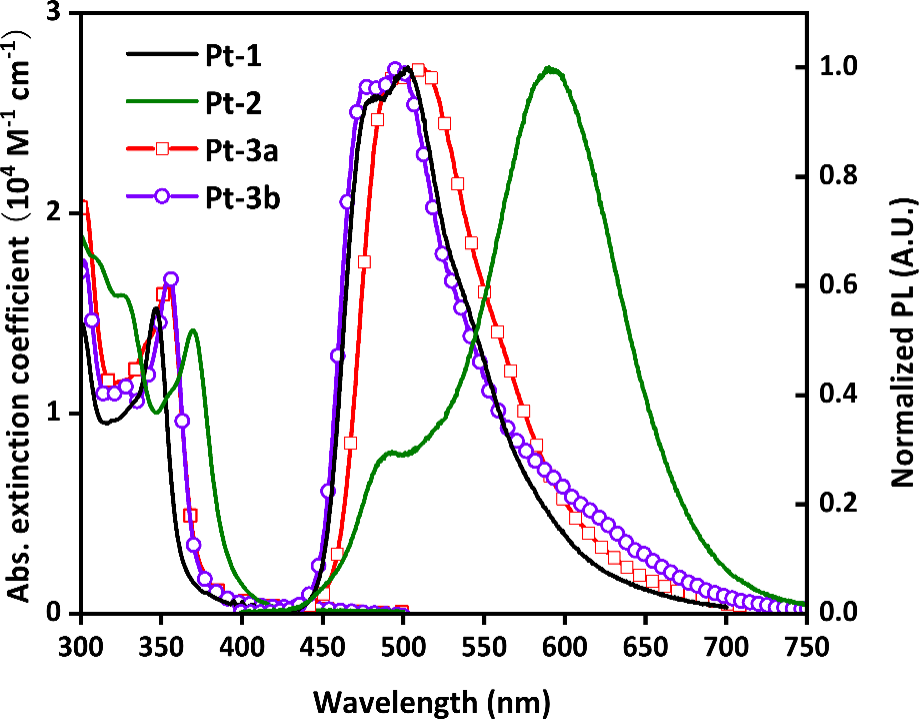
Absorption and photoluminescence spectra of studied Pt­(II) complexes in toluene with a conc. of 10^–5^ M at RT.

**Table 1:** Photophysical Data of the Studied Pt­(II) Complexes

	abs λ_max_ (nm)^a^	em λ_max_ (nm)^a^,^b^	fwhm (nm)^a^,^b^	PLQY (%)^c^,^d^	τ_obs_ (μs)^c^,^e^	τ_rad_ (μs)	*k*_r_ (10^5^ s^–1^)	*k*_nr_ (10^5^ s^–1^)
**Pt-1**	347 (1.52)	482, 502 [472,491]	84 [67]	69	3.97	5.75	1.74	0.78
**Pt-2**	369 (1.41)	490, 591 [484, 502, 575(sh)]	97 [129]	55	4.05	7.36	1.36	1.11
**Pt-3a**	354 (1.66)	494, 511 [481,498]	88 [70]	61	3.65	5.98	1.67	1.07
Pt-3b	355 (1.69)	478, 497 [467,486]	83 [68]	59	3.32	5.63	1.78	1.23

a Recorded in degassed toluene at a conc. of 10^–5^ M at RT; the absorption extinction coefficient (ε) is given in parentheses with a unit of 10^4^ M^–1^ cm^–1^.

b Data in square brackets were recorded in a 2 wt % PMMA film at RT; excitation wavelength λ_exc_ = 330 nm.

c Photoluminescence quantum yield and τ_obs_ were recorded in a 2 wt % PMMA film at RT.

d PLQY data were measured by an integration sphere.

e Data measured at a peak max. of ∼480 nm.

Next, the absorption and emission spectra of both **Pt-1** and **Pt-2** were measured with varied concentrations in toluene for further verification of this behavior. As shown in Figure 6, the **im** ^**i**^ **z**-substituted complex **Pt-1** exhibited no apparent change in both absorption and emission spectra over the concentration range of 1 × 10^–6^ to 5 × 10^–5^ M. As to the **fppz**-functionalized **Pt-2**, although it exhibited no visible change in absorption spectra, its emission spectra have changed from a narrow band at 464 nm at 1 × 10^–6^ M to a broadened dual emission with two peak max. at 484 and 587 nm at 5 × 10^–6^ M and, finally, turned to a broadened lower-energy band at 595 nm at 5 × 10^–5^ M. Hence, the lower-energy emission of **Pt-2** is not originated from the pre-existing dimer or aggregate due to the lack of the so-called metal–metal-to ligand charge transfer (MMLCT) band in absorption spectra but from a multistep process involving sequential formation of a single excited molecule (M*) and a bimolecular excimer (MM*), followed by giving excimer emission at the longer wavelength region.^64–67^ These transformations are depicted in eqs (i)−(iii):

$$\begin{array}{l} (i)\;M+h\nu \to M^{*}\text{;}\\ {(ii)\;M}^{*}+M\to {\mathrm{MM}}^{*}\text{;}\\ {(iii)\;\mathrm{MM}}^{*}\to 2M+h\nu^{\prime } \end{array}$$

**Figure 6:**
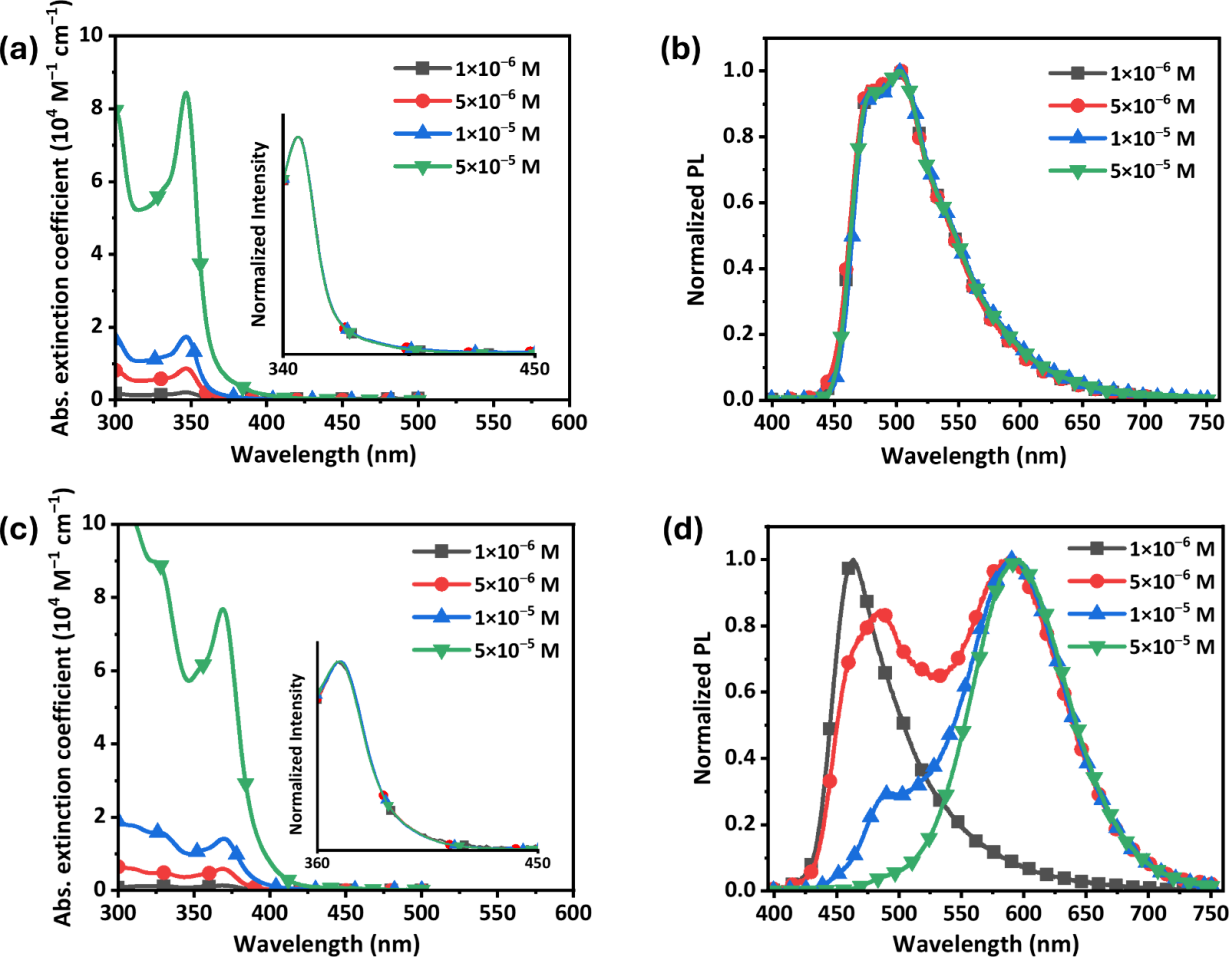
Absorption and emission spectra of (a, b) **Pt-1** and (c, d) **Pt-2** recorded in toluene at RT with indicated concentrations; normalized absorption spectra of both complexes are also inserted for direct comparison.

Nevertheless, formation of excimers on **Pt-1** is inhibited due to the bulky isopropyl substituent on imidazolyl chelate **im** ^**i**^ **z**. This revealed the effectiveness of peripheral groups in controlling aggregation and giving blueshifted emission.^9,68^

Finally, PMMA-doped thin films were prepared with varied concentrations of Pt­(II) complexes to demonstrate their emission pattern and performance in the solid state. Cast thin films with concentrations of 0.5, 2, 30, and 60 wt % were prepared by spin-coating from a mixed dichloromethane and PMMA solution (19:1), and the obtained photoluminescence spectra are shown in Figure 7. For both **Pt-1** and **Pt-3a**, the spectra resemble those obtained in toluene, with the vibrational fine structure being less well-resolved due to the higher polarity of the PMMA matrix. Most importantly, they showed only a minimal increase in emission intensity at the longer wavelength region at higher concentrations. This behavior is consistent with the results of single-crystal X-ray structural analysis, to which the intermolecular stacking interaction will be inhibited due to unfavorable packing motifs existing in the solid state. On the other hand, as the concentration of **Pt-2** and **Pt-3b** was increased to 30 wt % and higher, in addition to the original higher-energy band, a new band at a longer wavelength grows at λ_max_ = ∼582 and ∼566 nm, respectively. This concentration dependence is indicative of an increased contribution of emission from the bimolecular entity for **Pt-2** and the supramolecular aggregate for **Pt-3b** in both the solid state and doped thin film, which turned more dominant in comparison to the unimolecular emission occurring at the relative higher-energy region.^69–72^

**Figure 7:**
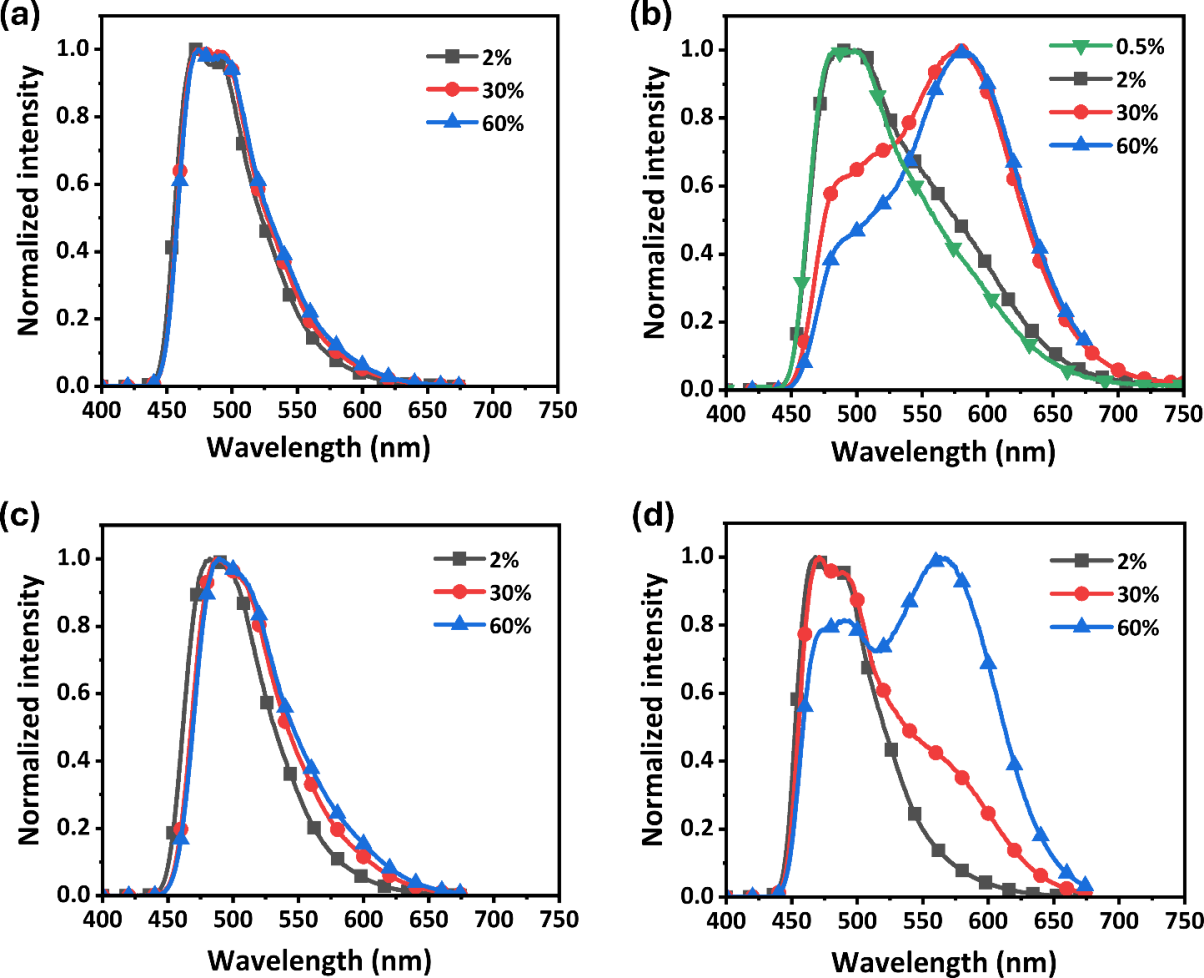
PL spectra of (a) **Pt-1**, (b) **Pt-2**, (c) **Pt-3a**, and (d) **Pt-3b**, dispersed in the PMMA matrix at RT with varied concentrations.

Cyclic voltammetry (CV) and thermogravimetric analysis were also conducted, see Table 2 and Figures S2 and S3. For the former, an irreversible oxidation peak was detected with the onset potential following two independent trends: **Pt-1** (*E*_ox_ = 0.70 V) < **Pt-2** (*E*_ox_ = 0.74 V) and **Pt-3a** (*E*_ox_ = 0.82 V) < **Pt-3b** (*E*_ox_ = 0.87 V). These variations agreed with the better electron-donating character of the imidazolyl vs pyridinyl coordination fragment between a pair of Pt­(II) complexes **Pt-1** and **Pt-2** and the higher electron-donating character of 4-t-butylphenyl vs phenyl cyclometalates for the second set of Pt­(II) complexes **Pt-3a**/**b**. Hence, the one with a higher electron density at the Pt­(II) metal center would exhibit lowered oxidation potentials. Moreover, their HOMO and LUMO energy levels were calculated using these oxidation potentials and the optical energy gaps estimated from the photophysical measurements, cf. Table 2. Concurrently, high decomposition temperatures (*T*_d_, at 5% weight loss) of 383, 406, 367, and 380 °C were recorded for **Pt-1**, **Pt-2**, **Pt-3a**, and **Pt-3b**, respectively. These photophysical and thermal data confirmed their readiness to serve as OLED devices.

**Table 2:** Electrochemical and Thermogravimetric Data of the Studied Pt­(II) Complexes

complex	*E*_ox,onset_ (eV)^a^	*E*_HOMO_ (eV)^b^	*E*_g_ (eV)^c^	*E*_LUMO_ (eV)^d^	*T*_d,5%_ (°C)^e^
**Pt-1**	0.70	–5.50	2.80	–2.70	383
**Pt-2**	0.74	–5.54	2.75	–2.78	406
**Pt-3a**	0.82	–5.62	2.77	–2.85	367
Pt-3b	0.87	–5.67	2.82	–2.85	380

a All electrochemical data were measured in an acetonitrile solution of TBAPF_6_ at 0.1 M. *E*_ox,onset_ is the onset potential of oxidation waves.

b HOMO = −(*E*_ox,onset_ + 4.8).

c Energy gap = 1240/[PL_onset_ (nm)].

d LUMO = HOMO + energy gap.

e TGA is recorded under a N_2_ atmosphere.

#### Theoretical Investigation

To elucidate how chelate modifications influence the electronic structures and photophysical properties of the Pt­(II) complexes, we carried out time-dependent density functional theory (TD-DFT) calculations^73–75^ and analyzed the electronic transitions associated with the S_1_ and T_1_ states. Further computational details are provided in the Supporting Information (SI).

TD-DFT calculations conducted on the optimized S_0_ structures in toluene yielded vertical S_0_ → S_1_ excitation energies for **Pt-1**, **Pt-2**, and **Pt-3a/b** at 383, 409, and 408/405 nm, respectively (Table 3). These results follow a consistent trend with the experimental absorption tails near 400 nm (Figure 5). The notably low oscillator strength (*f*) values for the S_0_ → S_1_ transition of **Pt-1** (0.0026), **Pt-2** (0.0012), and **Pt-3a/b** (0.0037/0.0022) indicate a weak excitation process. For the S_0_ → T_1_ transition at the S_0_ structures, the vertical excitation energies for **Pt-1**, **Pt-2**, and **Pt-3a/b** are 433, 454, and 456/446 nm, respectively, showing a mean absolute deviation (MAD) of ∼0.22 eV from the experimental emission peak max. λ_max_ (482, 490, and 494/478 nm, respectively, Table 1). In comparison, adiabatic emission energies derived from fully optimized T_1_ and S_0_ structures (482 nm for **Pt-1**, 491 nm for **Pt-2**, and 500/486 nm for **Pt-3a/b**, Table S1) agree more closely with experimental results, with a reduced MAD of ∼0.08 eV.

**Table 3:** TD-DFT Calculation Results and Molecular Orbital (MO) Contributions (>15%) for the S_0_ → T_1_ and S_0_ → S_1_ excitations^d^

						assignment^c^					
*E*_HOMO_/*E*_LUMO_(eV)^a^		excitation	λ [nm/eV]^b^	*f* ^b^	MO contribution (>15%)^b^	MLCT	LMCT	LLCT	LC	MC	
**Pt-1**	–5.43/–1.42	S_0_ → T_1_	433/2.86	0	HOMO → LUMO (84.0%)	18.1%	10.6%	15.1%	53.4%	2.8%	
		S_0_ → S_1_	383/3.23	0.0026	HOMO → LUMO (95.6%)						
**Pt-2**	–5.55/–1.86	S_0_ → T_1_	454/2.73	0	HOMO → LUMO (72.6%)	19.8%	7.5%	33.4%	37.2%	2.1%	
		S_0_ → S_1_	409/3.03	0.0012	HOMO → LUMO (94.1%)						
**Pt-3a**	–5.58/–1.80	S_0_ → T_1_	456/2.72	0	HOMO → LUMO (82.5%)	20.5%	10.0%	11.9%	54.5%	3.1%	
		S_0_ → S_1_	408/3.04	0.0037	HOMO → LUMO (95.2%)						
**Pt-3b**	–5.60/–1.79	S_0_ → T_1_	446/2.78	0	HOMO → LUMO (80.5%)	24.6%	8.6%	18.8%	44.7%	3.3%	
		S_0_ → S_1_	405/3.06	0.0022	HOMO → LUMO (94.8%)						

						assignment^c^					
*E*_HOMO_/*E*_LUMO_ (eV)		excitation	λ [nm/eV]^b^	*f* ^b^	MO contribution (>15%)^b^	MMLCT	LMMCT	LLCT	LC	MC	MMCT
**(Pt-3a)**_**2**_	–5.36/–1.84	S_0_ → T_1_	479/2.59	0	HOMO → LUMO (86.6%)	48.7%	6.5%	23.4%	12.5%	4.4%	4.4%
		S_0_ → S_1_	439/2.82	0.0315	HOMO → LUMO (93.4%)						
**(Pt-3a)**_**3**_	–5.29/–1.88	S_0_ → T_1_	487/2.55	0	HOMO → LUMO (75.7%)	51.1%	6.0%	20.4%	13.5%	3.9%	5.2%
		S_0_ → S_1_	452/2.74	0.0617	HOMO → LUMO (89.7%)						
**(Pt-3b)**_**2**_	–5.39/–1.80	S_0_ → T_1_	464/2.67	0	HOMO → LUMO (85.2%)	46.1%	6.9%	25.8%	12.8%	4.1%	4.1%
		S_0_ → S_1_	428/2.90	0.0255	HOMO → LUMO (92.9%)						
**(Pt-3b)**_**3**_	–5.27/–1.76	S_0_ → T_1_	464/2.67	0	HOMO → LUMO (81.2%)	63.6%	3.9%	15.7%	4.7%	4.1%	8.0%
		S_0_ → S_1_	434/2.86	0.0956	HOMO → LUMO (85.3%)						

a The *E*_HOMO_ and *E*_LUMO_ of the optimized S_0_ structures calculated at B3LYP-D3­(BJ)/def2-SVP level.

b The vertical excitation energy (λ), oscillator strength (*f*), and MO contribution obtained by the TD-DFT method at the B3LYP-D3­(BJ)/def2-SVP level.

c The assignments studied by the IFCT (Hirshfeld) method at the optimized S_0_ structures.

d The charge transfer character assignments for S_0_ → T_1_ excitation of the studied complexes of **Pt-1**, **Pt-2**, **Pt-3a**, and **Pt-3b** (the monomers were analyzed with PCM in toluene; the dimer and trimer complexes were studied in the gas phase) at their optimized S_0_ structures.

To further elucidate the S_0_ → T_1_ transitions in the studied Pt­(II) complexes, the natural transition orbital (NTO) analysis was performed based on the S_0_ structures.^76^ As illustrated in Figure 8, the occupied NTOs of all Pt­(II) complexes are primarily localized on both the Pt­(II) cation and carbene chelates (in π orbitals). In contrast, the virtual NTOs of **Pt-1** and **Pt-3a/b** resided predominantly on the carbene chelates (in π* orbitals), while those of **Pt-2** are extended across both the carbene and pyrazolate chelates. These patterns support a mixed metal-to-ligand charge transfer (MLCT) and ligand-centered (LC, i.e., intraligand transfer) character in all complexes, with **Pt-2** additionally exhibiting a significant ligand-to-ligand charge transfer contribution (LLCT, i.e., ligand-to-ligand charge transfer between the cyclometalating carbene and pyridyl pyrazolate chelate).

**Figure 8:**
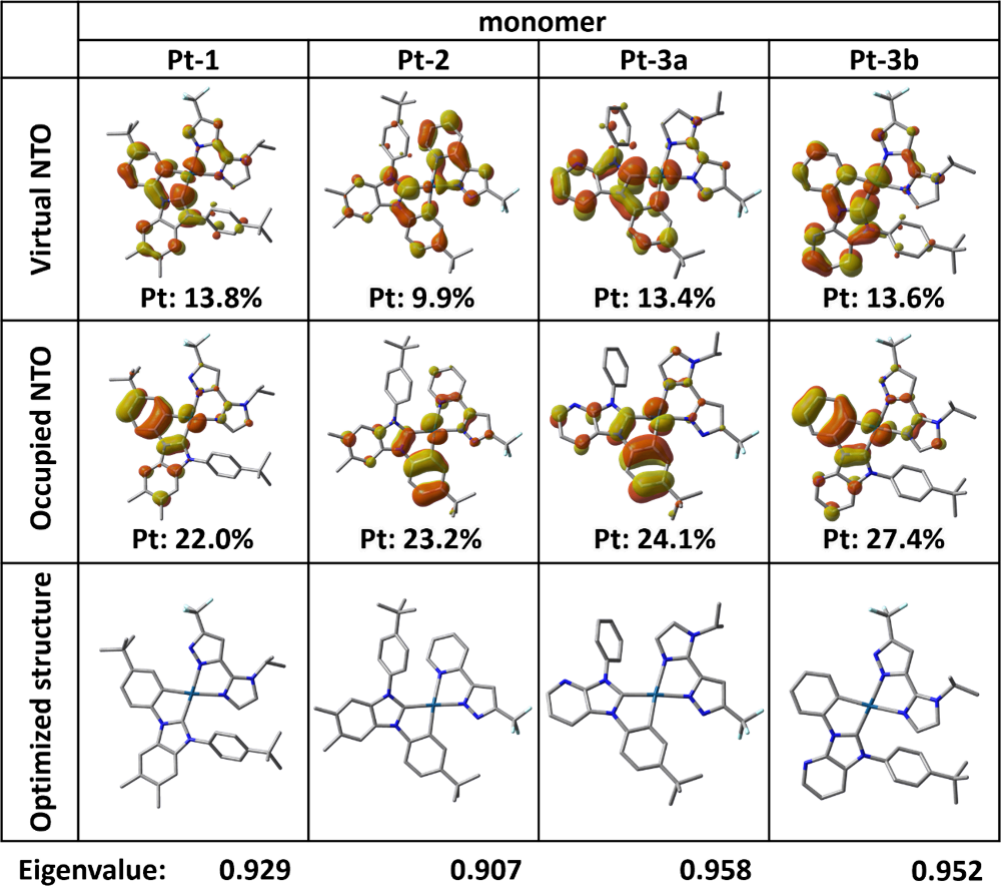
Dominant eigenvalues and natural transition orbital (NTO) pairs for S_0_ → T_1_ excitation based on the optimized S_0_ structures of the studied monomers with PCM in toluene, including the composition of the Pt­(II) center to the NTOs. Hydrogen atoms were omitted for clarity.

To quantify and examine the contributions of the transition contributions of MLCT, ligand-to-metal charge transfer (LMCT), LLCT, LC, and metal-centered (MC) characters, the interfragment charge transfer (IFCT) analysis of the S_0_ → T_1_ transition for all studied Pt­(II) complexes was performed by Multiwfn software based on their optimized S_0_ structures (Table 3).^77^ For **Pt-1**, **Pt-3a**, and **Pt-3b**, the MLCT and LC contributions are, respectively, 18.1 and 53.4%, 20.5 and 54.5%, and 24.6 and 44.7%, consistent with the NTO analysis. In **Pt-2**, mixed characteristics are observed, with moderate MLCT (19.8%), elevated LLCT (33.4%), and reduced LC (37.2%) contributions. In current DFT studies, correlating individual MLCT percentages with emission rates (*k*_r_) remains challenging, especially across structurally distinct complexes like **Pt-1** vs **Pt-2** vs **Pt-3a/b**. However, the trend among isomers usually supports that a higher MLCT component generally favors a faster *k*_r_. For instance, the higher MLCT proportion in **Pt-3b** (24.6%) compared to **Pt-3a** (20.5%) aligns with its slightly larger experimental *k*_r_ (1.78 × 10^5^ s^–1^ vs 1.67 × 10^5^ s^–1^).

The spin–orbit coupling (SOC)-TD-DFT method was next employed to estimate the τ_rad_ and *k*_r_ values of all the optimized S_0_ and T_1_ structures for the monomers.^78–81^ Both arithmetic and Boltzmann averages were used to calculate the results (Table S1); for the sake of brevity, only Boltzmann-averaged values are discussed. The calculated τ_rad_ values of T_1_ → S_0_ emission in **Pt-1** (4.06 μs) and **Pt-3a/b** (5.95/4.69 μs) at their optimized S_0_ structures correspond well with the experimental results (5.75 μs for **Pt-1** and 5.98/5.63 μs for **Pt-3a/b**; Table 1). For **Pt-2**, the predicted τ_rad_ values (3.71 μs at the S_0_ structure and 4.91 μs at the T_1_ structure) are moderately smaller than the experimental value (7.36 μs).

Motivated by the notable concentration dependence of **Pt-3a** and **Pt-3b**, we extended our TD-DFT calculations to their dimers and trimers in the gas phase. The vertical excitation energies for the S_0_ → T_1_ transition exhibit a distinct redshift in both the **Pt-3b** dimer and trimer (464 and 464 nm, respectively, Table 3) compared to that of the monomer (446 nm). Subsequent IFCT and NTO analyses (Figure 9) revealed a pronounced increase in the (M)­MLCT contribution (from 24.6% in the monomer to 46.1% in the dimer and 63.6% in the trimer). This progressive increase indicates strengthened Pt–Pt interactions in the aggregated species of **Pt-3b**, suggesting a remarkable tendency for this complex to form dimers, trimers, and even larger ordered assemblies. As for the **Pt-3a** dimer, it displayed a similarly elevated MMLCT percentage (48.7%, Table 3) like that of the **Pt-3b** dimer; however, a stable **Pt-3a** trimer configuration with unequal Pt···Pt distances is found instead; it featured irregular NTO distributions (Figure S4, Supporting Information) and notably asymmetric Pt···Pt distances (3.51/3.84 Å), in contrast with the symmetric, tight stacking structure observed in **Pt-3b** (3.65/3.69 Å, Figure 9). Furthermore, the MMLCT percentage for this **Pt-3a** “trimer” (51.1%) closely resembles that of its dimer, supporting the interpretation that this species represents a dimer loosely associated with a monomer rather than a genuine cooperatively stacked trimer.

**Figure 9:**
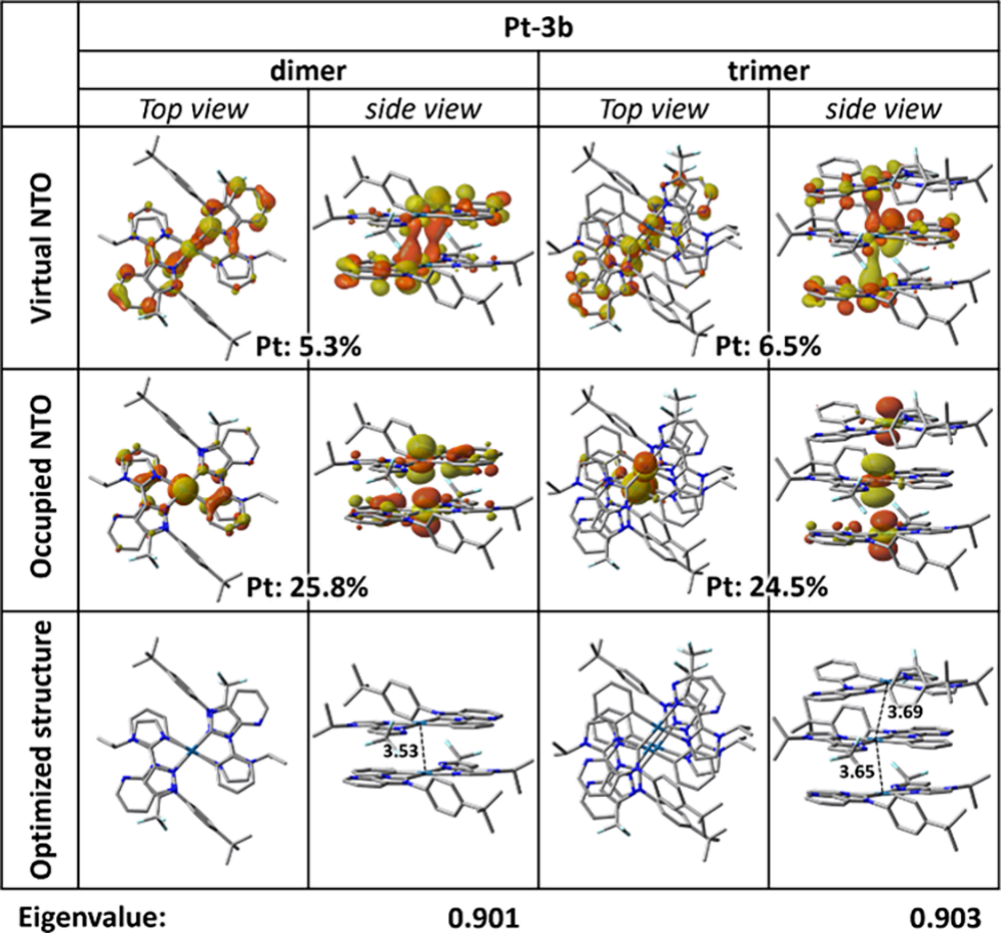
Dominant eigenvalues and NTO pairs for S_0_ → T_1_ excitation based on the optimized S_0_ structures of the selected **Pt-3b** dimer and trimer in the gas phase, including the composition of the Pt­(II) center to the NTOs. The Pt···Pt distances are given in Å, and the hydrogen atoms were omitted for clarity.

This divergent aggregation behavior stems from distinct steric profiles between the two isomers. **Pt-3a** experiences significant steric incompatibility arising from two structural features: a skewed noncoordinating ring of the carbene chelate combined with a *tert*-butyl group on the opposing coordinating ring. This steric congestion, exacerbated by the colocalization of CF_3_ and *tert*-butyl groups on the same side of the molecule, effectively prevents extended and ordered stacking interaction. In contrast, **Pt-3b** presents a more favorable steric profile, with its *tert*-butyl group positioned on the noncoordinating aryl group, and the opposing coordinating aryl group is free from the bulky substituent. In addition, the **Pt-3a** and **Pt-3b** monomers have similar stability, whereas the **Pt-3b** dimer and trimer are more stable than the **Pt-3a** dimer and trimer by 0.3–0.4 eV in the gas phase at the B3LYP-D3­(BJ)/def2-SVP level. These computational insights into their distinct aggregation propensities aligned well with the experimental observations.

##### OLED Fabrication and Characterization

To elucidate the influence of chelate modification on electro-optical properties, **Pt-1** and **Pt-2** were selected as representative complexes for an in-depth investigation, as **Pt-3a/b** exhibited similar photophysical properties to **Pt-1** but lower synthetic yields. The devices employed a cohost system comprising 1,3-bis­(*N*-carbazolyl)­benzene (mCP) and (1,3,5-triazine-2,4,6-triyl)­tris­(benzene-3,1-diyl)­tris­(diphenylphosphine oxide) (PO-T2T) in a 1:1 weight ratio for the emissive layer (EML). The device architecture was as follows: indium tin oxide (ITO)/mCP:rhenium oxide (ReO_3_) (24:1, 45 nm)/mCP (15 nm)/mCP:PO-T2T (1:1, 20 nm):*x* wt % **Pt-1** or **Pt-2**/PO-T2T (50 nm)/8-hydroxyquinolinolatolithium (Liq) (0.5 nm)/aluminum (Al) (100 nm), based on our previous report.^82^ To facilitate hole injection from the ITO anode to the mCP layer, ReO_3_ was introduced as a p-type dopant to form an ohmic contact.^83^ The hole-transporting layer (HTL) utilized high triplet energy mCP (*E*_T_ = 2.94 eV, HOMO/LUMO = 6.1/2.4 eV), while the electron-transporting layer (ETL) employed PO-T2T (*E*_T_ = 2.99 eV, HOMO/LUMO = 7.5/3.5 eV). These two materials concurrently formed a 1:1 exciplex cohost system within the EML.^84^ Liq and Al served as the electron-injection layer and cathode, respectively. The electroluminescence (EL) characteristics and key performance metrics of the devices featuring **Pt-1** and **Pt-2** are presented in Figure S5 and Figure 10, while their numeric data are summarized in Table 4.

**Figure 10:**
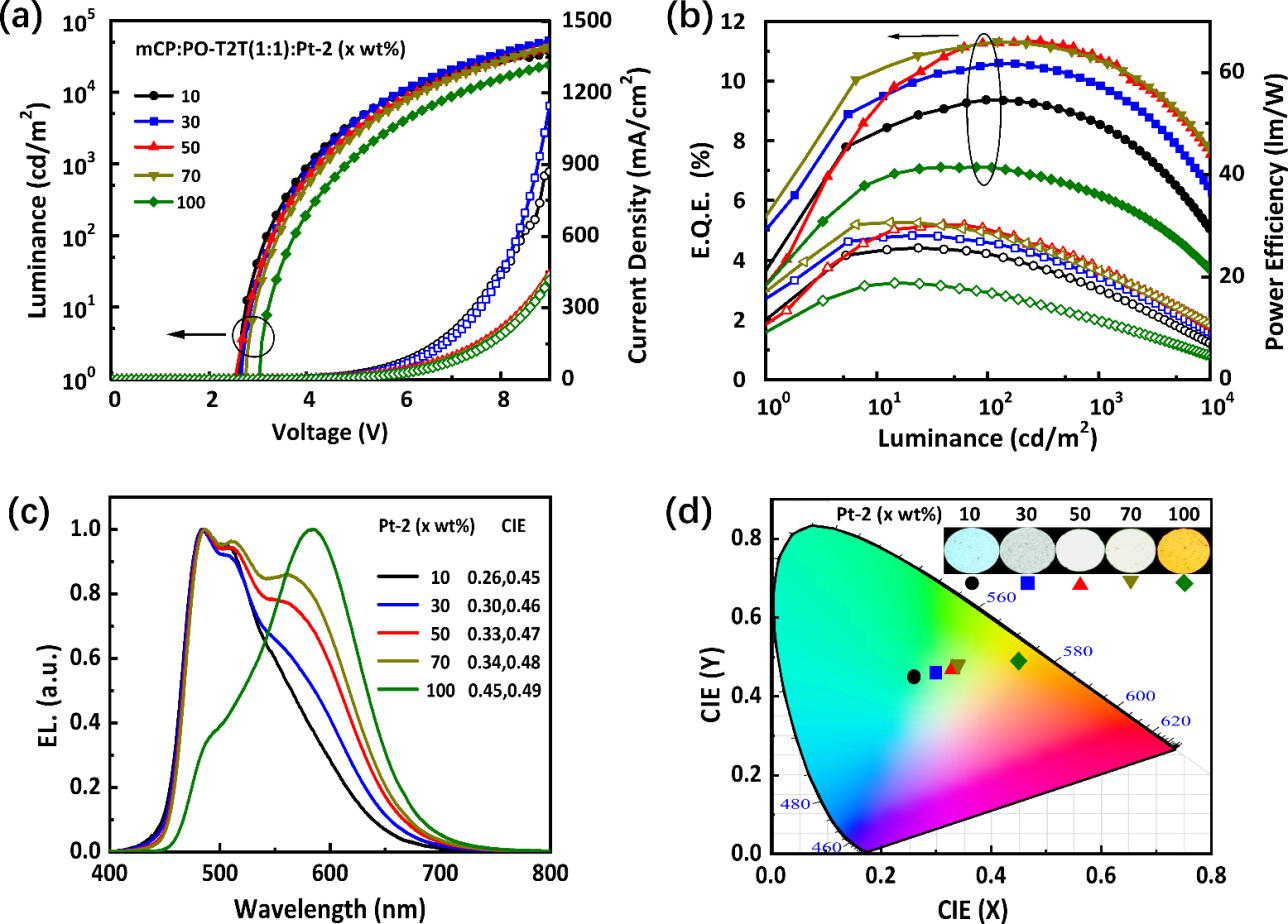
(a) Current density–voltage–luminance (*J*–*V*–*L*) characteristics, (b) external quantum (EQE) and power efficiencies (PE) as a function of luminance, (c) EL spectra, and (d) CIE_*xy*_ coordinates of **Pt-2**-based devices at various doping concentrations; the inset depicts the actual color of the OLED devices.

**Table 4:** Electroluminescence Data of the Fabricated OLED Devices

entries	*V*_on_ [V]	max. EQE/CE/PE [%, cd A^–1^, lm W^–1^]	data at 1000 cd m^–2^ [%, cd A^–1^, lm W^–1^, V]	roll-off [%]^a^	λ_max_ [nm]^b^	CIE_*xy*_ ^b^
**Pt-1**: 10 wt %	2.6	10.1/22.1/22.1	9.2/21.0/14.0/4.5	8.9	470	0.19, 0.34
30 wt %	2.6	11.1/24.3/22.5	10.4/22.8/16.3/4.4	6.3	470	0.20, 0.36
50 wt %	2.6	11.7/26.9/28.0	10.7/24.6/18.8/4.1	8.5	470	0.20, 0.37
**Pt-2**: 10 wt %	2.6	9.4/25.1/25.7	8.7/23.2/18.3/4.0	7.4	484	0.26, 0.45
30 wt %	2.6	10.6/28.5/28.1	10.0/26.9/20.6/4.1	5.7	484	0.30, 0.46
50 wt %	2.6	11.3/31.2/30.2	10.9/30.0/22.5/4.2	3.5	486	0.33, 0.47
70 wt %	2.7	11.3/31.5/30.6	10.8/30.0/22.0/4.3	4.4	486	0.34, 0.48
100 wt %	3.0	7.1/20.4/18.8	6.3/17.9/11.7/4.8	11.3	584	0.45, 0.49

a Efficiency roll-off recorded at 1000 cd m^–2^.

b Data measured at 100 cd m^–2^.

The EL spectra of **Pt-1**-based OLEDs were found to be largely independent of the dopant concentration, a behavior consistent with its photoluminescence (PL) emission profiles (Figures 6 and 7). Specifically, the EL spectrum remained nearly invariant across a concentration range from 10 to 50 wt % in doped thin films, exhibiting stable sky-blue emission characterized by two vibronic peak max. at 470 and 496 nm (Figure S5). The best performance was achieved at a doping concentration of 50 wt %, delivering a maximum external quantum efficiency (max. EQE) of 11.7%, a max. current efficiency (max. CE) of 26.9 cd A^–1^, and a max. power efficiency (max PE) of 27.8 lm W^–1^. Notably, all of the OLED devices exhibited a small efficiency roll-off of ∼10% at 1000 cd m^–2^. This feature is not only attributed to a broadened charge recombination zone and a balanced carrier transporting property within the EML but also due to the relatively shortened radiative lifetime of **Pt-1** (5.75 μs), which suppressed triplet–triplet annihilation (TTA) and triplet-polaron annihilation (TPA) under high luminance.

In contrast to **Pt-1**, the EL spectra of **Pt-2**-based OLEDs were highly dependent on the doping concentration, as depicted in Figure 10. Upon increasing the **Pt-2** concentration (from 10 to 100 wt %), a distinct excimer emission at the longer wavelength emerged. Its intensity also increased at the higher doping ratio, which is akin to its PL recorded in toluene solution or PMMA thin films.^35^ As can be seen, the device produced sky-blue emission with a structured EL profile and its peal max. located at 484 and 508 nm, giving CIE_*xy*_ of (0.26, 0.45) at 10 wt %. As the doping ratio was further increased to 50 wt %, the excimer emission at 584 nm was significantly enhanced, and hence, the color turned to white with CIE_*xy*_ of (0.33, 0.47). In the meantime, there exists an improvement in efficiency but with little efficiency roll-off, for example, the max. EQE was improved from 9.4 to 11.3%, and the efficiency roll-off was kept at only 3.5%. Finally, when a neat film of **Pt-2** (100 wt %) was employed as the EML, the device generated yellow emission with CIE_*xy*_ of (0.45, 0.49) and with max. EQE, PE, and CE of 7.1%, 20.4 cd A^–1^, and 18.8 lm W^–1^, respectively. Figure S6 shows the color shifting of **Pt-2**-based devices under varied conditions. They consisted of mixed high-energy monomer emission (∼484 nm) and low-energy excimer emission (∼584 nm), with the perceived color being determined by their relative intensities. As the driving voltage increased, the low-energy excimer emission began to saturate and excess energies are then captured by the high-energy monomers. As a result, the short-wavelength monomer emission grows notably, causing CIE_*xy*_ coordinates to shift toward the blue region of the EL spectrum.

Alternatively, the excimer emission of the **Pt-1** complex is effectively suppressed by the intrinsic steric hindrance introduced by the isopropyl substituent of imidazolyl pyrazolate. This finding underscores the efficacy of utilizing sterically demanding peripheral groups to mitigate solid-state aggregation in maintaining high-energy, monomeric blue emission. Therefore, **Pt-1** performs as a stable and efficient blue phosphor, while **Pt-2** behaves as a concentration-sensitive dopant, enabling facile tuning of the emission from blue to yellow by modulating the monomer-to-excimer emission ratio, i.e., the doping concentration in the light-emitting layer.

## Conclusions

In summary, four Pt­(II) complexes featuring both the carbene cyclometalate (C^C) and chelating pyrazolate (N^N), namely, **Pt-1**, **Pt-2**, and **Pt-3a/b**, were successfully designed and prepared. There are two feasible synthetic pathways: the first employed the monodentate carbene complex **Pt-DMSO** as an intermediate, followed by addition of the N^N chelate in giving **Pt-1-Cl** and then aryl cyclometalation in giving **Pt-1**, while the second involved consecutive addition of C^C and N^N chelates to the Pt­(II) metal reagent Pt­(COD)­Cl_2_ for **Pt-2**, among which we preferred the second due to its milder operational conditions. Moreover, those with an asymmetric carbene chelate, i.e., **Pt-3a** and **Pt-3b**, provided a unique platform in revealing facile interchanging of the N-aryl cyclometalate via Ir–C bond cleavage and retro-C-H activation, as shown by efficient and reversible interconversion between these isomeric products.

Next, their photophysical properties were probed in both degassed toluene and the doped PMMA matrix at RT, revealing great dependence on their coordinative chelates. To those with carbene chelate **LA** (i.e., with two *N*-4-*t*-butylphenyl appendages), Pt­(II) complex **Pt-1** featuring an **im** ^**i**^ **z** chelate showed concentration-independent photoluminescence under all conditions vs **Pt-2** with the **fppz** chelate, showing the notable effect of the *i*-propyl group of the **im** ^**i**^ **z** chelate in inhibiting the intermolecular stacking interaction and, hence, giving both the suppressed excimer emission in solution and MMLCT emission in the solid state. In sharp contrast, Pt­(II) complex **Pt-2** exhibited mainly the lower-energy excimer emission at a higher concentration due to the lack of a bulky group on the **fppz** chelate. Moreover, for the pairs of Pt­(II) complexes **Pt-3a/b** with asymmetric carbene chelate **LB**, the coordinated 4-butylphenyl cyclometalate of **Pt-3a** exerted greater steric encumbrance against intermolecular stacking interaction vs **Pt-3b** with the less congested phenyl cyclometalate, to which the latter exhibited notable lower-energy emission in both solution and solid states at high concentrations. These aggregation propensities aligned well with the TD-DFT calculations based on the dimers and trimers of **Pt-3a/b** in the gas phase.

Lastly, the OLED devices based on **Pt-1** exhibited a concentration-invariant emission profile with a max. EQE of 11.7% and CIE_*xy*_ of (0.20, 0.36) at 50 wt %, while OLED devices based on **Pt-2** gave concentration-dependent emission with max. EQE of 9.4, 11.3, and 7.1% and CIE_*xy*_ of (0.26, 0.45), (0.33, 0.47), and (0.45, 0.49) at doping concentrations of 10, 50, and 100 wt %, respectively. Hence, these results demonstrated new strategies in designing color-tunable Pt­(II) emitters and associated OLED devices.

## Experimental Section

### General Information and Materials

All reactions were conducted under a N_2_ atmosphere. Commercially available reagents were used without further purification, and solvents were dried prior to use. No uncommon hazards are noted. ^1^H and ^19^F NMR spectra were measured with a Bruker Avance NEO 400 MHz NMR spectrometer, and ^195^Pt NMR spectra were measured with a Bruker Avance III 600 MHz NMR spectrometer. The high-resolution mass spectra were obtained on a Sciex X500R Q-TOF, whereas acetonitrile was applied as the solvent. The nitrogen chelates, namely, 5-(1-isopropyl-1*H*-imidazol-2-yl)-3-(trifluoromethyl)-1*H*-pyrazole **(im** ^**i**^ **z)­H** and 2-(3-(trifluoromethyl)-1*H*-pyrazol-5-yl)­pyridine **(fppz)­H**, were prepared in accordance with literature methods.^35,36^

### Synthesis of Pt­(II) Complex **Pt-1-DMSO**

To a 100 mL flask were added **LAH**_**2**_^+^ (560 mg, 1 mmol), sodium acetate (410 mg, 5 mmol), Pt­(DMSO)_2_Cl_2_ (420 mg, 1.5 mmol), and a mixture of acetone and DMSO (v/v = 5/1) (60 mL), and then, the mixture was refluxed for 2 h. After cooling to RT, the solvent was removed under reduced pressure, and the residue was dissolved in ethyl acetate and then washed with deionized water. The organic phase was separated, dried over anhydrous Na_2_SO_4_, and concentrated under reduced pressure. The residue was purified by a flash column with a mixture of *n*-hexane and ethyl acetate (v/v = 5/1) to afford a crude product of **Pt-1-DMSO**. Further recrystallization from a mixture of CH_2_Cl_2_ and hexane attained a white solid of **Pt-1-DMSO** (450 mg, 60%).

Spectroscopic data of **Pt-1-DMSO**. HRMS (ESI) for [M + H]^+^: calcd., 754.1959; found, 754.1968; ^1^H NMR (400 MHz, CDCl_3_): δ 7.78 (d, br, *J* = 7.6 Hz, 4H), 7.66 (d, *J* = 8.8 Hz, 4H), 7.04 (s, 2H), 2.88 (s, *J*_Pt–H_ = 10.2 Hz, 6H), 2.32 (s, 6H), 1.43 (s, 18H).

### Synthesis of Pt­(II) Complex **Pt-1-Cl**

To a 50 mL flask were added **Pt-1-DMSO** (377 mg, 0.5 mmol), sodium acetate (205 mg, 2.5 mmol), **(im^i^z)­H** (122 mg, 0.5 mmol), and DMSO (20 mL), and then, the mixture was heated at 120 °C for 4 h. After cooling to RT, DMSO was removed under vacuum, and the residue was dissolved in ethyl acetate and then washed with deionized water. It was separated, dried over anhydrous Na_2_SO_4_, and concentrated under reduced pressure. The residue was purified by column chromatography with a mixture of *n*-hexane/ethyl acetate/CH_2_Cl_2_ (v/v/v = 4/1/1) to afford a crude product of **Pt-1-Cl**. Further recrystallization from a mixture of CH_2_Cl_2_ and methanol attained a white solid of **Pt-1-Cl** (397 mg, 90%).

Spectroscopic data of **Pt-1-Cl**. HRMS (ESI) for [M + H]^+^: calcd., 884.2989; found, 884.2980; ^1^H NMR (400 MHz, CDCl_3_): δ 7.70 (d, *J* = 8.0 Hz, 4H), 7.46 (d, *J* = 8.0 Hz, 4H), 7.13 (s, 1H), 7.02 (s, 1H), 6.88 (s, 2H), 6.43 (s, 1H), 4.55 (m, 1H), 2.31 (s, 6H), 1.46 (s, 3H), 1.44 (s, 3H), 1.34 (s, 18H). ^19^F NMR (376 MHz, CDCl_3_): δ −60.44 (s, 3F). ^195^Pt NMR (129 MHz, CDCl_3_): δ −3159.

Selected crystal data of **Pt-1-Cl**. CCDC deposition number: 2429133. C_40_H_48_ClF_3_N_6_OPt; *M* = 916.38; monoclinic; space group = *P*2_1_/*n*; *a* = 10.7177(18) Å, *b* = 17.752(3) Å, and *c* = 20.957(3) Å; β = 97.575(6)°; *V* = 3952.4(11) Å^3^; *Z* = 4; ρ_Calcd_ = 1.54 g cm^–3^; μ = 3.671 mm^–1^; *F*(000) = 1840.0, λ­(Mo–Kα) = 0.71073 Å; *T* = 243(2) K; crystal size: 0.34 × 0.19 × 0.02 mm; 83,219 reflections collected, 9805 independent reflections (*R*_int_ = 0.1034), data/restraints/parameters = 9805/360/547, GOF = 1.047, final *R*_1_ [*I* > 2σ­(*I*)] = 0.0432, and *w*R_2_ (all data) = 0.0958.

### Synthesis of Pt­(II) Complex **Pt-1**

#### Method A

To a 50 mL flask were added **Pt-1-Cl** (177 mg, 0,2 mmol), sodium acetate (82 mg, 1 mmol), and *o-*dichlorobenzene (20 mL), and then, the mixture was refluxed for 72 h. After removal of the solvent under vacuum, the residue was dissolved in ethyl acetate and washed with deionized water. It was next dried over anhydrous Na_2_SO_4_ and concentrated under reduced pressure. The crude product was further purified by flash column chromatography and recrystallization from a mixture of CH_2_Cl_2_ and methanol to attain a white solid of **Pt-1** (156 mg, 92%).

#### Method B

To a 100 mL flask were added **LAH**_**2**_^+^ (560 mg, 1 mmol), sodium acetate (410 mg, 5 mmol), Pt­(COD)­Cl_2_ (376 mg, 1 mmol), and DMF (40 mL), and the mixture was heated at 80 °C overnight. After that, the imidazolyl chelate **(im** ^**i**^ **z)­H** (244 mg, 1 mmol) was added, and the mixture was heated at 120 °C for another 4 h. After removal of DMF under vacuum, the residue was dissolved in ethyl acetate and washed with deionized water. The organic phase was next separated, dried over anhydrous Na_2_SO_4_, and concentrated under reduced pressure. The residue was purified by column chromatography with a mixture of *n*-hexane/ethyl acetate (v/v = 3/1) to afford a crude product of **Pt-1**. Further recrystallization from a mixture of CH_2_Cl_2_ and methanol attained a light-yellow solid of **Pt-1** (400 mg, 47%).

Spectroscopic data of **Pt-1**. HRMS (ESI) for [M + H]^+^: calcd., 848.3222; found, 848.3227; ^1^H NMR (400 MHz, CDCl_3_): δ 9.79–9.56 (d, *J* = 2.0 Hz, *J*_Pt–H_ = 29.2 Hz, 1H), 7.84 (s, 1H), 7.71 (d, *J* = 8.4 Hz, 2H), 7.58–7.48 (m, 3H), 7.23 (dd, *J* = 8.0, 1.6 Hz, 1H), 7.13 (s, 1H), 6.62 (s, 1H), 6.39 (s, 1H), 4.89 (s, 1H), 4.68–4.57 (m, 1H), 2.44 (s, 3H), 2.31 (s, 3H), 1.48 (s, 9H), 1.41 (s, 3H), 1.39 (s, 3H), 1.38 (s, 9H). ^19^F NMR (376 MHz, CDCl_3_): δ −60.61 (s, 3F). ^195^Pt NMR (129 MHz, CDCl_3_): δ −3836.

Selected crystal data of **Pt-1**. CCDC deposition number: 2429138. C_40.5_H_44.5_F_3_N_6_PtCl_4.5_; *M* = 1026.93; triclinic; space group = $P\overset{®}{1}$ ; *a* = 11.3637(5) Å, *b* = 11.4161(4) Å, and *c* = 16.9835(6) Å; α = 80.0100(10)°, β = 87.0950(10)°, and γ = 86.458(2)°; *V* = 2163.91(14) Å^3^; *Z* = 2; ρ_Calcd_ = 1.576 g cm^–3^; μ = 3.569 mm^–1^; *F*(000) = 1022.0, λ­(Mo–Kα) = 0.71073 Å; *T* = 213(2) K; crystal size: 0.41 × 0.17 × 0.02 mm; 24,135 reflections collected, 8838 independent reflections (*R*_int_ = 0.0785), data/restraints/parameters = 8838/54/524, GOF = 1.011, final *R*_1_ [*I* > 2σ­(*I*)] = 0.0503, and *wR*_2_ (all data) = 0.1243.

### Synthesis of Pt­(II) Complex **Pt-2**

To a 100 mL flask were added **LAH**_**2**_^+^ (249 mg, 0.5 mmol), sodium acetate (205 mg, 2.5 mmol), Pt­(COD)­Cl_2_ (188 mg, 0.5 mmol), and DMF (40 mL) and then heated at 80 °C overnight. Next, pyridyl chelate **(fppz)­H** (106 mg, 0.5 mmol) was added and the mixture was heated at 120 °C for another 4 h. After cooling to RT, DMF was removed under vacuum, and the residue was dissolved in ethyl acetate and washed with deionized water three times. The organic phase was separated, dried over anhydrous Na_2_SO_4_, and concentrated under reduced pressure. The residue was purified by column chromatography and eluted with a mixture of *n*-hexane and ethyl acetate (v/v = 5/1). Further recrystallization from a mixture of CH_2_Cl_2_ and methanol attained a light-green solid of **Pt-2** (237 mg, 58%).

Spectroscopic data of **Pt-2**. HRMS (ESI) for [M + H]^+^: calcd., 817.2800; found, 817.2826; ^1^H NMR (400 MHz, CDCl_3_): δ 9.57 (d, *J* = 2.0 Hz, *J*_Pt–H_ = 29.2 Hz, 1H), 8.54 (dd, *J* = 6.0, 3.2 Hz, 1H), 8.47 (dd, *J* = 4.8, 1.2 Hz, 1H), 7.73 (d, *J* = 8.4 Hz, 2H), 7.67 (dd, *J* = 8.0, 1.2 Hz, 1H), 7.53 (d, *J* = 8.4 Hz, 2H), 7.28–7.17 (m, 2H), 6.60 (s, 1H), 6.37 (d, *J* = 1.6 Hz, 1H), 4.85 (d, *J* = 1.6 Hz, 1H), 4.68–4.54 (m, 1H), 1.43 (s, 3H), 1.41 (s, 3H), 1.39 (s, 9H). ^19^F NMR (376 MHz, CDCl_3_): δ −60.34 (s, 3F). ^195^Pt NMR (129 MHz, CDCl_3_): δ −3700.

Selected crystal data of **Pt-2**. CCDC deposition number: 2469397. C_38_H_38_F_3_N_5_Pt; *M* = 816.82; monoclinic; space group = *P*2_1_; *a* = 12.7578(4) Å, *b* = 30.4316(11) Å, and *c* = 18.1237(5) Å; β = 91.3280(10)°; *V* = 7034.5(4) Å^3^; *Z* = 8; ρ_Calcd_ = 1.543 g cm^–3^; μ = 4.039 mm^–1^; *F*(000) = 3248.0, λ­(Mo–Kα) = 0.71073 Å; *T* = 173(2) K; crystal size: 0.37 × 0.14 × 0.02 mm; 172,769 reflections collected, 28,982 independent reflections (*R*_int_ = 0.0763), data/restraints/parameters = 28982/367/1792, GOF = 1.028, final *R*_1_ [*I* > 2σ­(*I*)] = 0.0325, and *wR*_2_ (all data) = 0.0758.

### Synthesis of Pt­(II) Complexes **Pt-3a/b**

To a 100 mL flask were added **LBH**_**2**_^+^ (475 mg, 1 mmol), sodium acetate (410 mg, 5 mmol), Pt­(COD)­Cl_2_ (376 g, 1 mmol), and DMF (40 mL) and then heated at 80 °C overnight. Next, imidazolyl chelate **(im^i^z)­H** (244 mg, 1 mmol) was added, and the mixture was heated at 120 °C for another 4 h. After removal of DMF under vacuum, the residue was dissolved in ethyl acetate and washed with deionized water. The organic phase was separated, dried over anhydrous Na_2_SO_4_, and concentrated under reduced pressure. The residue was purified by column chromatography with a mixture of *n*-hexane/ethyl acetate (3/1, v/v) to afford **Pt-3a** (*R*_f_ = 0.3) and **Pt-3b** (*R*_f_ = 0.7). Further recrystallization from a mixture of CH_2_Cl_2_ and methanol attained a light-green solid of **Pt-3a** (46 mg, 6%) and a yellow solid of **Pt-3b** (183 mg, 24%).

Spectroscopic data of **Pt-3a**. HRMS (ESI) for [M + H]^+^: calcd., 765.2235; found, 765.2263; ^1^H NMR (400 MHz, CDCl_3_): δ 9.77 (d, *J* = 2.0 Hz, *J*_Pt–H_ = 28.4 Hz, 1H), 8.43 (dd, *J* = 4.8, 1.2 Hz, 1H), 8.36 (dd, *J* = 8.0, 1.2 Hz, 1H), 8.03–7.94 (m, 2H), 7.59–7.50 (m, 3H), 7.48 (d, *J* = 8.0 Hz, 1H), 7.39 (dd, *J* = 8.0, 4.8 Hz, 1H), 7.23 (dd, *J* = 8.0, 2.0 Hz, 1H), 6.66 (s, 1H), 6.54 (d, *J* = 1.6 Hz, 1H), 5.03 (d, *J* = 1.6 Hz, 1H), 4.74–4.59 (m, 1H), 1.48 (s, 9H), 1.46 (s, 3H), 1.44 (s, 3H). ^19^F NMR (376 MHz, CDCl_3_): δ −60.72 (s, 3F). ^195^Pt NMR (129 MHz, CDCl_3_): δ −3784.

Selected crystal data of **Pt-3a**. CCDC deposition number: 2429140. C_32_H_30_F_3_N_7_Pt; *M* = 764.72; monoclinic; space group *P*2_1_/*n*; *a* = 11.8198(10) Å, *b* = 13.6577(12) Å, and *c* = 17.7745(15) Å; β = 92.365(2)°; *V* = 2866.9(4) Å^3^; *Z* = 4; ρ_Calcd_ = 1.772 g cm^–3^; μ = 4.95 mm^–1^; *F*(000) = 1504.0, λ­(Mo–Kα) = 0.71073 Å; *T* = 218(2) K; crystal size: 0.41 × 0.22 × 0.03 mm; 17,805 reflections collected, 5813 independent reflections (*R*_int_ = 0.0632), data/restraints/parameters = 5813/0/393, GOF = 1.015, final *R*_1_ [*I* > 2σ­(*I*)] = 0.0373, and *wR*_2_ (all data) = 0.0939.

Spectroscopic data of **Pt-3b**. HRMS (ESI) for [M + H]^+^: calcd., 765.2235; found, 765.2248; ^1^H NMR (400 MHz, CDCl_3_): δ 9.39 (dd, *J* = 6.0, 3.2 Hz, *J*_Pt–H_ = 28.4 Hz, 1H), 8.54 (dd, *J* = 6.0, 3.2 Hz, 1H), 8.47 (dd, *J* = 4.8, 1.2 Hz, 1H), 7.73 (d, *J* = 8.4 Hz, 2H), 7.67 (dd, *J* = 8.0, 1.2 Hz, 1H), 7.53 (d, *J* = 8.4 Hz, 2H), 7.28–7.17 (m, 3H), 6.60 (s, 1H), 6.37 (d, *J* = 1.6 Hz, 1H), 4.85 (d, *J* = 1.6 Hz, 1H), 4.68–4.54 (m, 1H), 1.43 (s, 3H), 1.41 (s, 3H), 1.39 (s, 9H). ^19^F NMR (376 MHz, CDCl_3_): δ −60.34 (s, 3F). ^195^Pt NMR (129 MHz, CDCl_3_): δ −3774.

Selected crystal data of **Pt-3b**. CCDC deposition number: 2429139. C_33_H_31_F_3_N_7_PtCl_3_; *M* = 884.09; triclinic; space group $P\overset{®}{1}$ ; *a* = 11.0122(4) Å, *b* = 12.7404(4) Å, and *c* = 12.7958(5) Å; α = 73.1240(10)°, β = 81.068(2)°, and γ = 83.5760(10)°; *V* = 1692.86(11) Å^3^; *Z* = 2; ρ_Calcd_ = 1.734 g cm^–3^; μ = 4.433 mm^–1^; *F*(000) = 868.0, λ­(Mo–Kα) = 0.71073 Å; *T* = 243(2) K; crystal size: 0.35 × 0.22 × 0.03 mm; 36,095 reflections collected, 8415 independent reflections (*R*_int_ = 0.0659), data/restraints/parameters = 8415/174/468, GOF = 1.020, final *R*_1_ [*I* > 2σ­(*I*)] = 0.0363, and *wR*_2_ (all data) = 0.0846.

### Isomerization of **Pt-3a** (or) **Pt-3b**

To a 25 mL flask were added **Pt-3a** (or **Pt-3b**) (100 mg, 0.131 mmol), TsOH·H_2_O (12 mg, 0.065 mmol), sodium acetate (107 mg, 1.31 mmol), and 1,2,4-trichlorobenzene (8 mL). The mixture was refluxed at 214 °C for 12 h with vigorous stirring. After being cooled to RT, the solvent was removed, and the residue was taken into CH_2_Cl_2_ and washed with deionized water. The organic phase was separated, dried over anhydrous Na_2_SO_4_, and concentrated under reduced pressure. The residue was purified by column chromatography with a mixture of *n*-hexane/ethyl acetate (v/v = 3/1) to afford approximately 75 mg of **Pt-3a** (17%) and 15 mg of **Pt-3b** (83%) for both attempts.

## Supplementary Material



## Notes

Synthetic procedures and spectroscopic data of chelates and associated intermediates; TD-DFT investigations, selected analytical data, and nonessential OLED data of studied Pt­(II) emitters (PDF)
