# Supplementary material for: Modulation of Supramolecular Interaction of Pt(II) Complexes Bearing Carbene Cyclometalate for Color-Tunable Luminescence
Source: Inorg Chem. 2025 Dec 3;64(49):24274–88. doi: 10.1021/acs.inorgchem.5c04898 (PMC12709578; doi:10.1021/acs.inorgchem.5c04898)
Supplement: Supplementary file 1 [file ic5c04898_si_001.pdf]

Supporting Information

Modulation of Supramolecular Interaction of Pt(II) Complexes Bearing Carbene Cyclometalate for Color-Tunable Luminescence

Yixin Wu,[a,†] Lin Cheng,[a,†] Yu-Cheng Kung,[b,†] Yi Pan,[a] Shek-Man Yiu,[a] Jie Yan,[a] Kai Li,[c,*] Wen-Yi Hung,[b,*] Yun Chi,[a,*] Kai Chung-Lau,[a,*]

[†] Y. Wu, L. Cheng and Y-C. Kung contributed equally to this work.

(a) Department of Chemistry, Department of Materials Science and Engineering, and Center of Super-Diamond and Advanced Films (COSDAF), City University of Hong Kong, Kowloon 999077, Hong Kong SAR, E-mail: yunchi@cityu.edu.hk, and E-mail: kaichung@cityu.edu.hk.
(b) Department of Optoelectronics and Materials Technology, National Taiwan Ocean University, Keelung 20224, Taiwan, E-mail: wenhung@mail.ntou.edu.tw.
(c) Guangdong Provincial Key Laboratory of New Energy Materials and Service Safety, Shenzhen Key Laboratory of New Information Display and Storage Materials, College of Materials Science and Engineering, Shenzhen University, Shenzhen 518071, China. E-mail: kaili@szu.edu.cn

Experimental section:

General information and materials. All reactions were conducted under N_2 atmosphere. Commercially available reagents were used without further purification and solvents were dried prior to use. ^1H and ^{19}F NMR spectra were measured with Bruker Avance NEO 400 MHz NMR spectrometer. The high-resolution mass spectra were obtained on Sciex X500R Q-TOF, whereas acetonitrile was applied as the solvent.

Photophysical measurements: UV-Vis spectra were recorded on a HITACHI UH-4150 spectrophotometer. The steady-state emission spectrum was measured with Edinburgh FS 980. Both wavelength-dependent excitation and emission responses of the fluorimeter were calibrated. The lifetime studies were performed by a time-correlated single photon counting system (TCSPC). Spectral grade solvents (Merck) were used as received. The photoluminescence quantum yields in solid state were measured by integrated sphere.

Electrochemistry: Cyclic voltammetry was conducted on a CHI621A Electrochemical Analyzer. Ag/Ag^+ (0.01 M $AgNO_3$) electrode was employed as reference electrode. The oxidation and reduction potentials were measured using a glassy carbon working electrode with 0.1 M of NBu_4PF_6 in CH_3CN. The potentials were referenced externally to the ferrocenium/ferrocene (Fc^+/Fc) couple.

Computational details of theoretical investigations: The geometries, electronic structures, and electronic excitations of the studied Pt(II) complexes were investigated at the B3LYP-D3(BJ)/def2-SVP level[1-4] using Gaussian 16 set of programs. [5] The solvent effect of toluene was taken account by the polarizable continuum model (PCM). [6-7] The structures of the studied Pt(II) complexes were optimized based on their X-ray crystallographic data of **Pt-1**, **Pt-2** and **Pt-3a/b**. Subsequently, the TD–DFT calculations[8-9] were performed based on their optimized S_0 structures, including the $T_1 \sim T_{100}$ and $S_1 \sim S_{100}$ excited states (200 states in total). For excitations involving multiple orbital contributions (e.g., $S_0 \rightarrow T_1$ excitation in this work), the natural transition orbital (NTO) analysis was employed. [10] The density in the IFCT analysis and the contribution of Pt(II) metal to the NTO pairs were quantified using the Hirshfeld method in Multiwfn. [11-12]

The spin-orbit coupling (SOC)-TDDFT computations[13] were executed in ORCA (v 6.0.1) software[14-15] at the optimized S_0 and T_1 structures using the B3LYP functional with ZORA Hamiltonian[16-17] (SARC-ZORA-SVP for Pt; ZORA-def2-SVP for other elements). A total of 200 low-lying excited states (100 for singlet and 100 for triplet) were within the COSMO solvation model for toluene. [18] The radiative lifetime (τ_{rad}) and radiative rate (k_r) were derived from the arithmetic average and Boltzmann average (at 298 K) of the SOC substates of the T_1 excited states. [12]

OLED fabrication and Characterization: Before fabricating the OLEDs, all compounds were subjected to purification via vacuum sublimation. The OLED devices were assembled by depositing materials onto glass substrates coated with indium tin oxide (ITO) at a pressure of 10^{-6} torr, with the substrates having a sheet resistance of 15 Ω sq^{-1}. The ITO surface was meticulously cleaned using an ultrasonic method, which involved sequential treatments with acetone, methanol, and deionized

water, concluding with a N_2 plasma treatment. Each organic material was deposited at a rate of about 1–2 Å·s^{-1}. The devices' J–V–L (current–voltage–luminance) characteristics were assessed in a glove box environment. A programmable source measurement unit (model Keithley 2614B) served as the driving source for the devices, while the light intensity was captured using a silicon detector that had been calibrated for accuracy. For spectral analysis, each electroluminescence (EL) spectrum was collected using an optical fiber connected to a spectrometer (Instrument Systems CAS 125).

Syntheses and characterization of employed carbene chelates:

Preparation of carbene chelate LAH_2^+

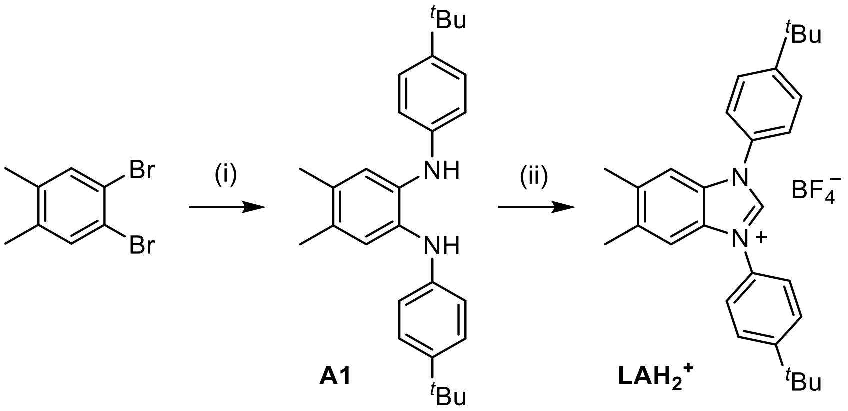

Scheme S1. Synthetic protocol to pro-chelate **LAH_2^+**; experimental conditions: 1,2-dibromo-4,5-dimethylbenzene, $Pd(OAc)_2$, 1,3-bis(2,6-diisopropylphenyl)imidazolium chloride, NaO^{t}Bu, toluene, 90 °C; (ii) trimethyl orthoformate, conc. HCl(aq), reflux.

Synthesis of A1

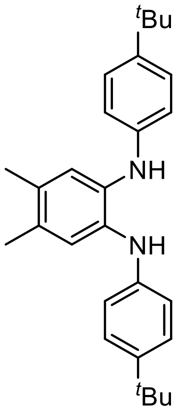

To a 50 mL flask was added $Pd(OAc)_2$ (10 mg, 0.045 mmol), 1,3-bis(2,6-diisopropylphenyl)imidazolium chloride (39 mg, 0.09 mmol), NaO^{t}Bu (0.48 g, 5.0 mmol) and degassed toluene (15 mL). The mixture was stirred for 10 minutes. After that, 1,2-dibromo-4,5-dimethylbenzene (0.6 g, 2.3 mmol) and 4-tert-butyl aniline (0.77 mL, 4.8 mmol) were added. The reaction mixture was then heated to 90 °C for 4 hours. After removal of the solvent under reduced

pressure, the residue was dissolved in ethyl acetate and filtered through Celite. The solution was then washed with deionized water, dried over anhydrous Na_2SO_4, concentrated and further purified by column chromatography with a mixture of hexane and ethyl acetate (20/1, v/v) to give a white solid **A1** (0.57 g, 63%).

Selected spectroscopic data of **A1**: ^{1}H NMR (400 MHz, $CDCl_3$) δ 7.26 (d, J = 8.4 Hz, 4H), 7.10 (s, 2H), 6.87 (d, J = 8.4 Hz, 4H), 5.46 (s, 2H), 2.20 (s, 6H), 1.32 (s, 18H).

Synthesis of LAH_2^+

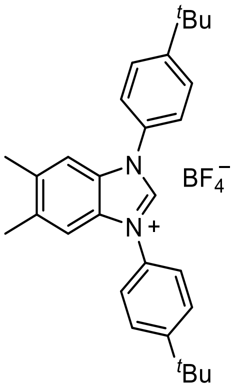

A1 (0.3 g, 0.75 mmol), trimethyl orthoformate (10 mL) and 1 drop of conc. $HCl_{(aq)}$ were added to a 50 mL flask. The reaction mixture was heated to reflux overnight and then, the triethyl orthoformate was removed under reduced pressure. The residue was washed with deionized water and ethyl acetate and then dried under vacuum to give a white product **LAH₂**$^+$ (3.71 g, 80%).

Selected spectroscopic data of **LAH₂**$^+$: ^{1}H NMR (400 MHz, DMSO-d_6) δ 10.30 (s, 1H), 7.87 – 7.80 (m, 8H), 7.77 (s, 2H), 2.46 (s, 6H), 1.41 (s, 18H). ^{19}F NMR (376 MHz, DMSO-d_6) δ –150.21 (^{10}B-F), –150.26 (^{11}B-F).

Preparation of carbene chelate LBH_2^+

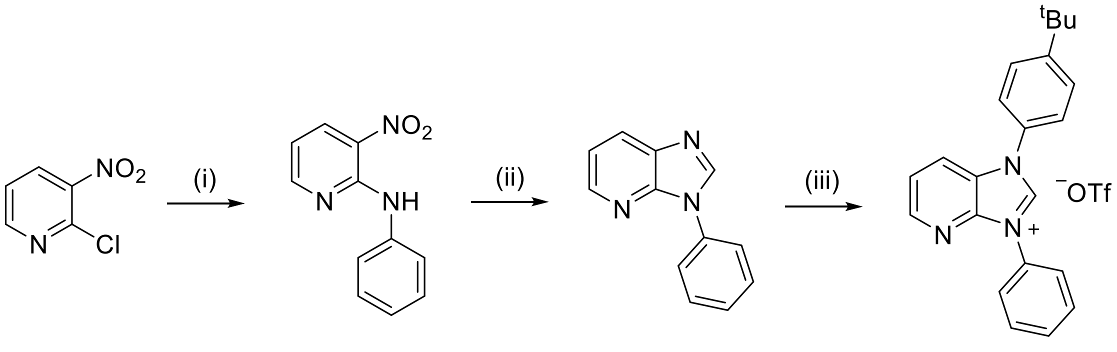

B1 **B2** **LBH₂⁺**

Scheme S2. Synthetic protocol to pro-chelate **LBH₂**$^+$; experimental conditions: (i) aniline, NEt_3, isopropanol/THF, reflux; (ii) Fe(s), HCO_2H, reflux; (iii) (4-^{t}Bu$C_6H_4I^+$Mes)(OTf^-), Cu_2O, DMF, 110 °C.

Synthesis of B1

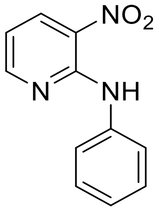

2-chloro-3-nitropyridine (6.32 g, 40 mml) was dissolved in a mixture of isopropanol and THF (100 mL, 1/1, v/v) in a 250 mL flask. After then, aniline (5.58 g,60 mmol) and Et_3N (6.06 g, 60 mmol) were added, and the mixture was heated to reflux for 12 hours. After cooling to RT, the mixture was concentrated to dryness and dissolved with ethyl acetate. The solution was washed with distilled water (100 mL × 3) and the organic layer was dried over anhydrous Na_2SO_4, filtered and concentrated to dryness to attain a red solid (**B1**) (7.3 g, 85 %).

Selected spectroscopic data of **B1**: ^1H NMR (400 MHz, $CDCl_3$) δ 10.13 (s, 1H), 8.53 (dd, J = 8.4, 1.6 Hz, 1H), 8.49 (dd, J = 4.4, 1.6 Hz, 1H), 7.65 (d, J = 7.6 Hz, 2H), 7.40 (t, J = 7.6, 2H), 7.19 (t, J = 7.6 Hz, 1H), 6.84 (dd, J = 8.4, 4.4 Hz, 1H).

Synthesis of B2

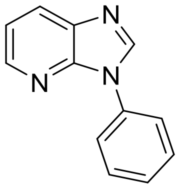

A mixture of **B1** (7.31 g, 34 mmol) and Iron power (11.4 g, 204 mmol) in formic acid (150 mL) was heated to reflux for 24 hours. Formic acid was removed under vacuum, and the residue was dissolved in ethyl acetate. The solution was filtered through celite, and the filtrate was washed with distilled water (100 mL ×3). The organic layer was dried over anhydrous Na_2SO_4, filtered and concentrated to dryness to afford a white solid (**B2**) (4.5 g, 68%).

Selected spectroscopic data of **B2**: ^1H NMR (400 MHz, $CDCl_3$) δ 8.49 (dd, J = 4.6, 1.6 Hz, 1H), 8.37 (s, 1H), 8.19 (dd, J = 8.0, 1.6 Hz, 1H), 7.78 (d, J = 7.6 Hz, 2H), 7.61 (t, J = 7.6 Hz, 2H), 7.48 (t, J = 7.6 Hz, 1H), 7.35 (dd, J = 8.0, 4.6 Hz, 1H).

Synthesis of LBH_2^+

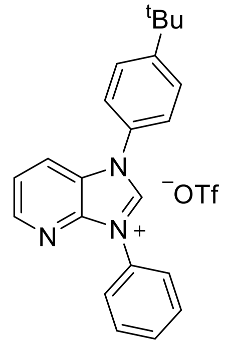

A mixture of **B2** (1.56 g, 8 mmol), (4-^{t}BuC$_6$H$_4$I^{+}Mes)(OTf^{-}) (3.78 g, 8.8 mmol) and Cu_2O (57 mg, 0.4 mmol) in anhydrous DMF (25 mL) was heated at 110 °C for 8 h. After cooling to RT, DMF was removed under vacuum and ethyl acetate (15 mL) was added to dissolve the crude product. The mixture was filtered through celite, and the filtrate was dried under reduced pressure to attain a grey solid. It was further triturated with ethyl acetate (5 mL). The resulting precipitate was filtered and dried under vacuum to give a white solid (3.1 g, 80%).

Selected spectroscopic data of **LBH$_2^+$**: ^{1}H NMR (400 MHz, $CDCl_3$) δ 10.15 (s, 1H), 8.83 (d, J = 4.0 Hz, 1H), 8.20 (d, J = 8.4 Hz, 1H), 8.05 (d, J = 7.2 Hz, 2H), 7.81 (d, J = 8.4 Hz, 2H), 7.77 – 7.68 (m, 3H), 7.67 – 7.57 (m, 3H), 1.40 (s, 9H). ^{19}F NMR (376 MHz, $CDCl_3$) δ –77.76 (s, 3F).

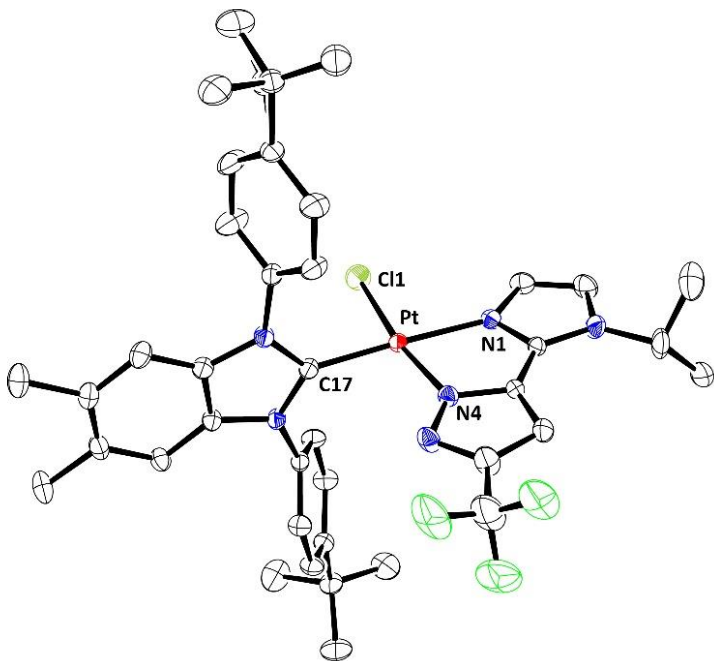

Figure S1. Structure drawing of **Pt-1-Cl** with thermal ellipsoids shown at 30% probability level. Selected bond length (Å): Pt-C(17) = 1.971(5), Pt-N(4) = 2.016(4), Pt-N(1) = 2.061(4), Pt-Cl(1) = 2.2936(14). Selected bond angle (°): N(4)-Pt-N(1) = 78.68(16), C(17)-Pt-Cl(1) = 89.10(15), C(17)-Pt-N(1) = 177.80(17), N(4)-Pt-Cl(1) = 171.28(12). Hydrogen atoms were omitted for clarity.

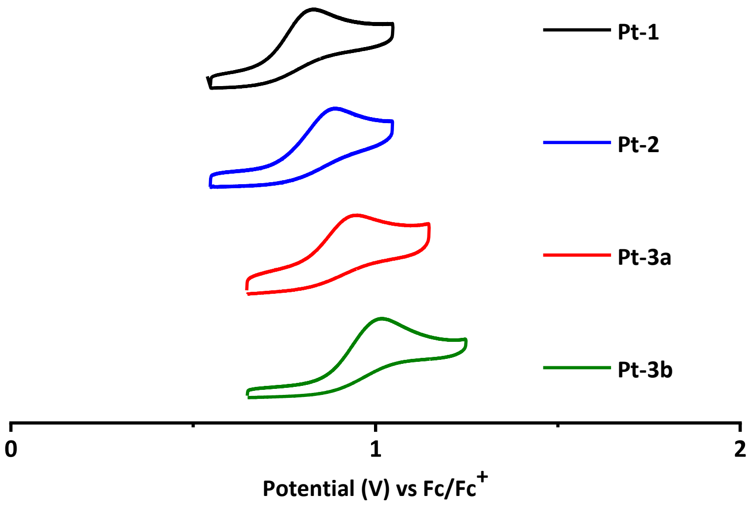

Figure S2. Cyclic voltammograms of studied Pt(II) complexes in acetonitrile solution.

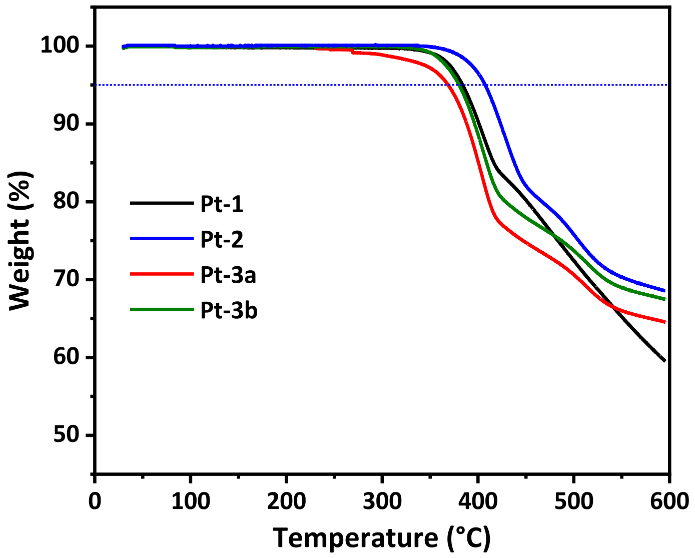

Figure S3. Thermogravimetric diagrams of studied Pt(II) complexes measured under N_2 atmosphere.

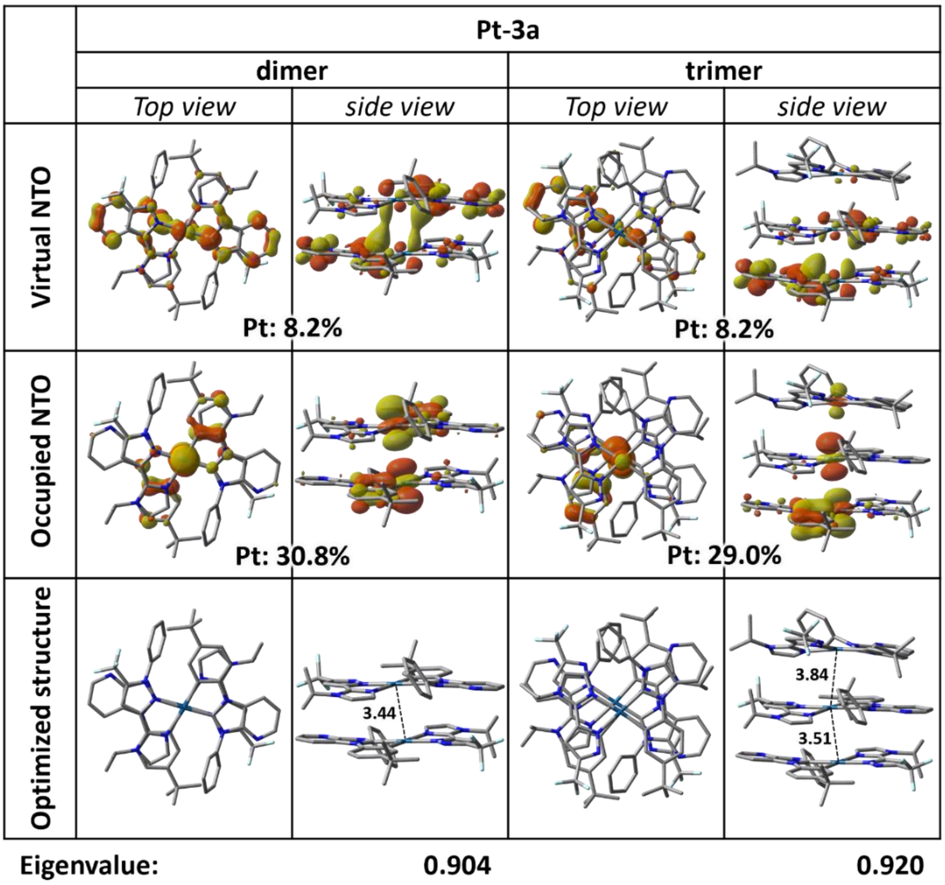

Figure S4. The dominant eigenvalues and NTO pairs for $S_0 \rightarrow T_1$ excitation based on the optimized S_0 structures of the selected **Pt-3a** dimer and trimer in the gas phase, including the composition of the Pt(II) center to the NTOs. The Pt···Pt distances are in Å and the hydrogen atoms were omitted for clarity.

Table S1. The predicted adiabatic emission energy of $T_1 \rightarrow S_0$ transition, emission radiative lifetime (τ_{rad}), and radiative rate (k_r) for all the studied complexes (the monomers were analysed with PCM in toluene; the dimer and trimer complexes were studied in the gas phase).

Emission ($T_1 \rightarrow S_0$)	λ [(a)] [nm/eV]	τ_{rad} [(b)] (μs)	k_r [(b)] (10^5 s^{-1})
**Pt-1**	482/2.57	3.85/4.06 (***5.33/5.34***)	2.60/2.46 (***1.88/1.87***)
**Pt-2**	491/2.53	3.58/3.71 (***4.88/4.91***)	2.80/2.69 (***2.05/2.04***)
**Pt-3a**	500/2.48	5.57/5.95 (***14.9/15.1***)	1.80/1.68 (***0.67/0.66***)
**Pt-3b**	486/2.55	4.15/4.69 (***3.82/3.86***)	2.41/2.13 (***2.62/2.59***)
**$(Pt\text{-}3b)_2$**	536/2.31	1.13/1.40 (***2.64/3.20***)	8.84/7.16 (***3.78/3.13***)
**$(Pt\text{-}3b)_3$**	539/2.30	0.79/1.01 (***2.30/2.61***)	12.6/9.91 (***4.34/3.82***)

(a) The computed adiabatic emission energy of the optimized T_1 and S_0 structures at B3LYP-D3(BJ)/def2-SVP level with zero-point energy correction.
(b) The calculated τ_{rad} and k_r are the arithmetic average/Boltzmann average (298 K) for the SOC substates at optimized S_0 (normal font) and T_1 (italic and bold font in the parentheses) structures at B3LYP-D3(BJ)/def2-SVP level.

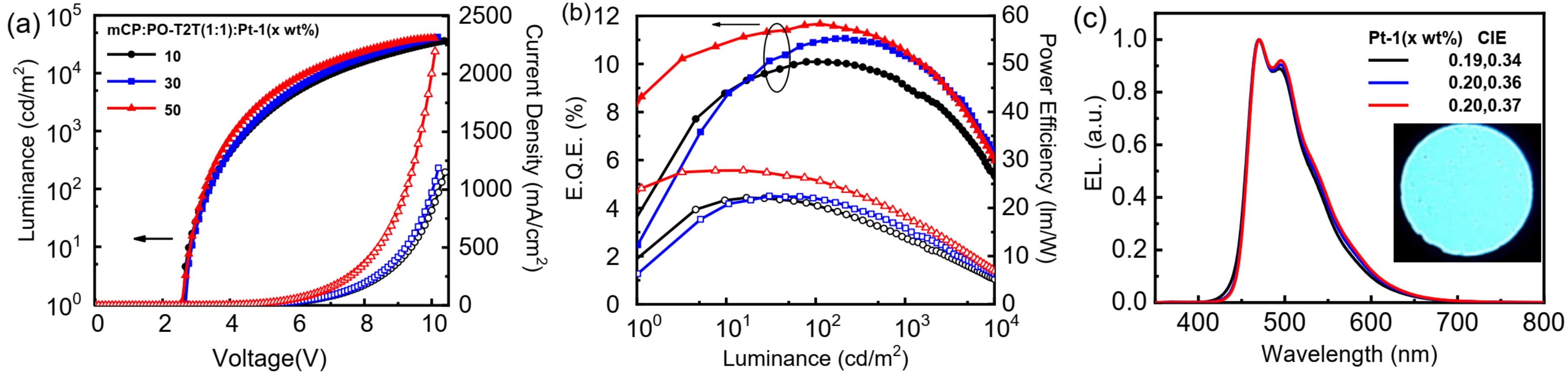

Figure S5. (a) Current density−voltage−luminance (J−V−L) characteristics, (b) external quantum (EQE) and power efficiencies (PE) as a function of luminance, and (c) EL spectra of **Pt-1**-based devices at various doping concentrations; inset depicts the photograph of OLED devices.

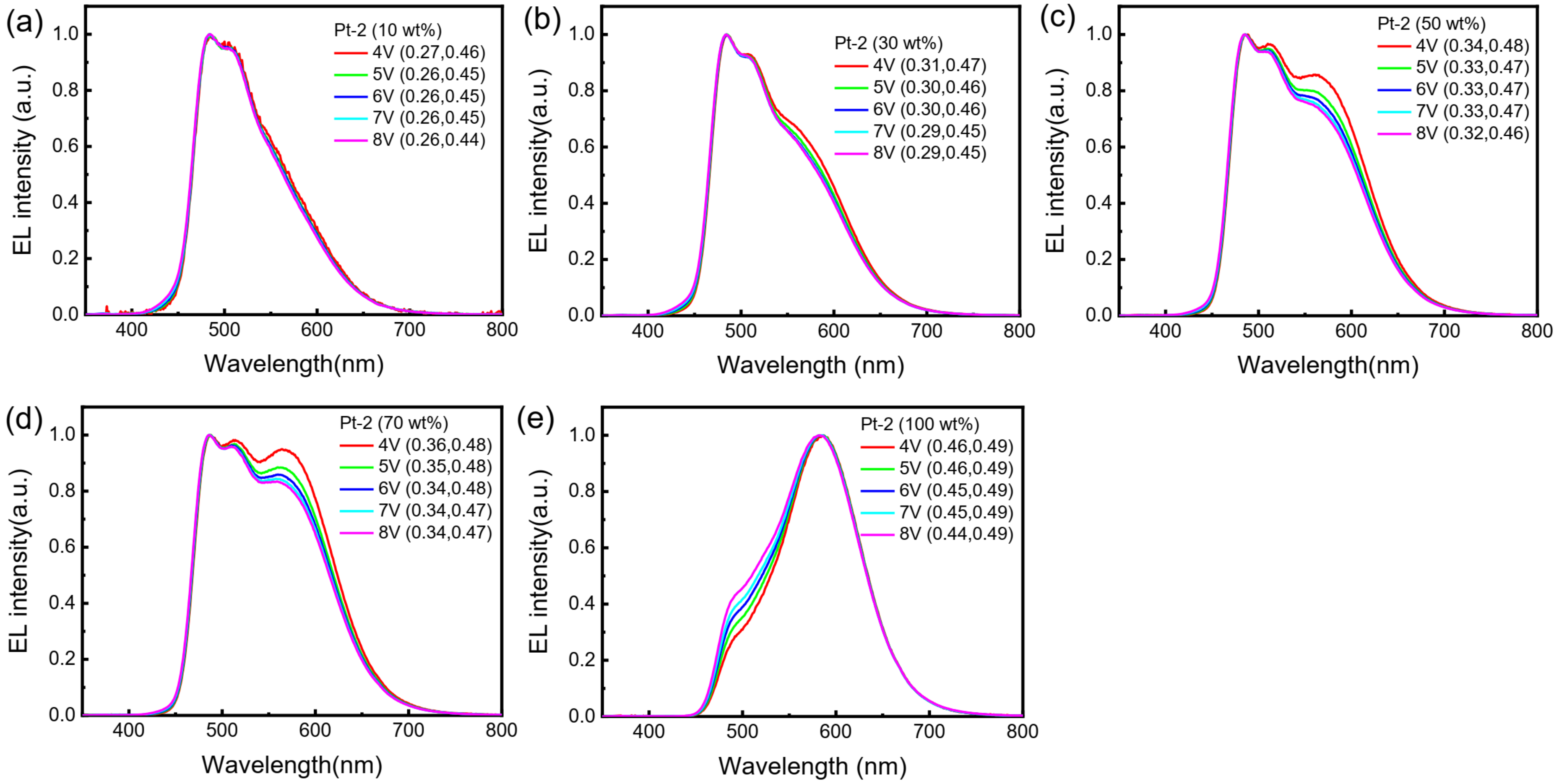

Figure S6. EL spectra of **Pt-2**-based devices under varied operational conditions.

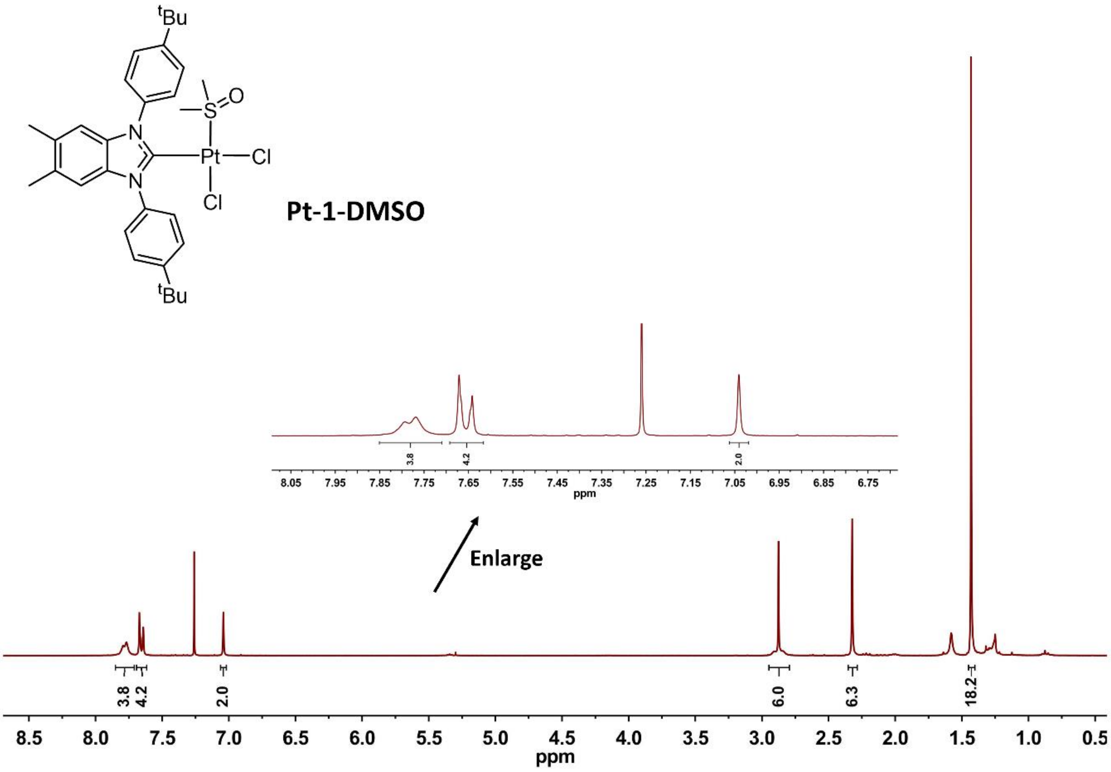

Figure S7. ^{1}H NMR spectrum (400 MHz) of **Pt-1-DMSO** in $CDCl_3$ at 298 K.

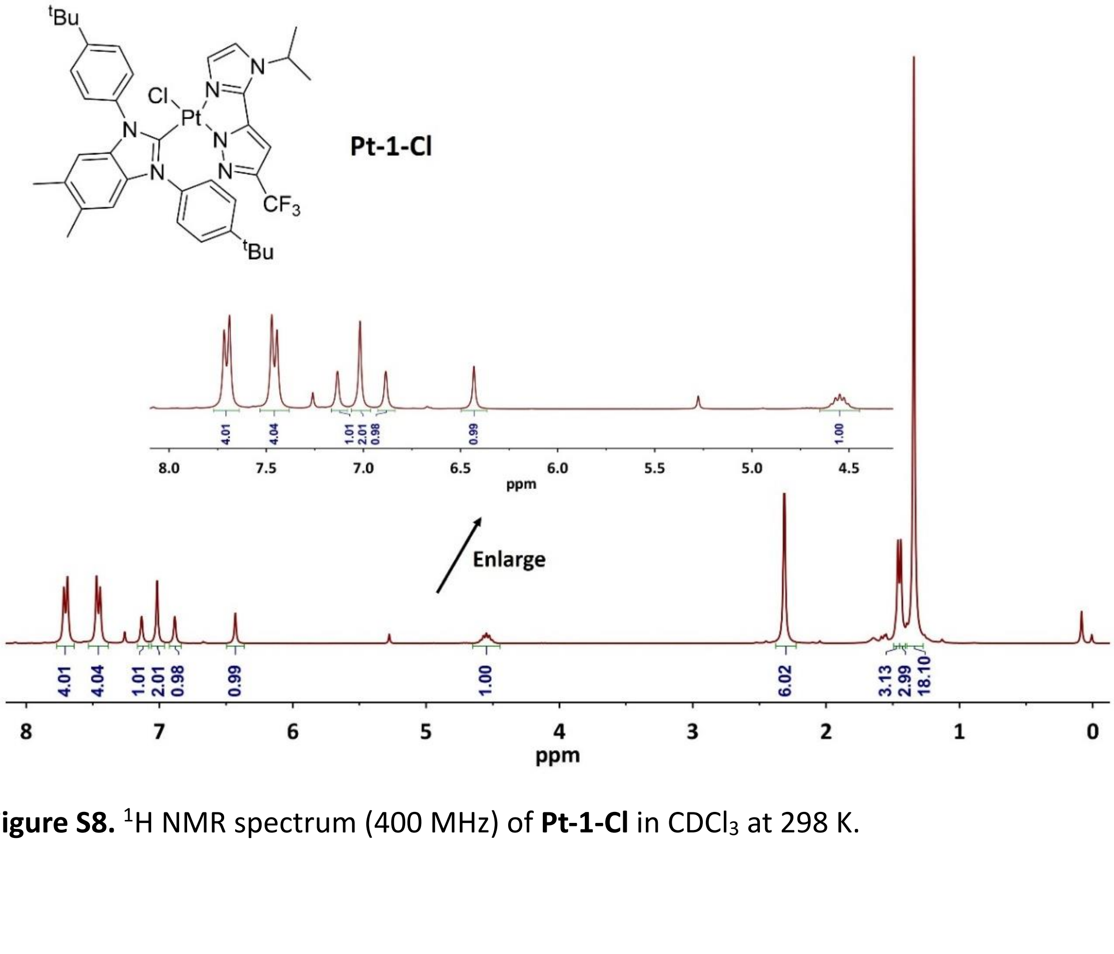

Figure S8. ^{1}H NMR spectrum (400 MHz) of **Pt-1-Cl** in $CDCl_3$ at 298 K.

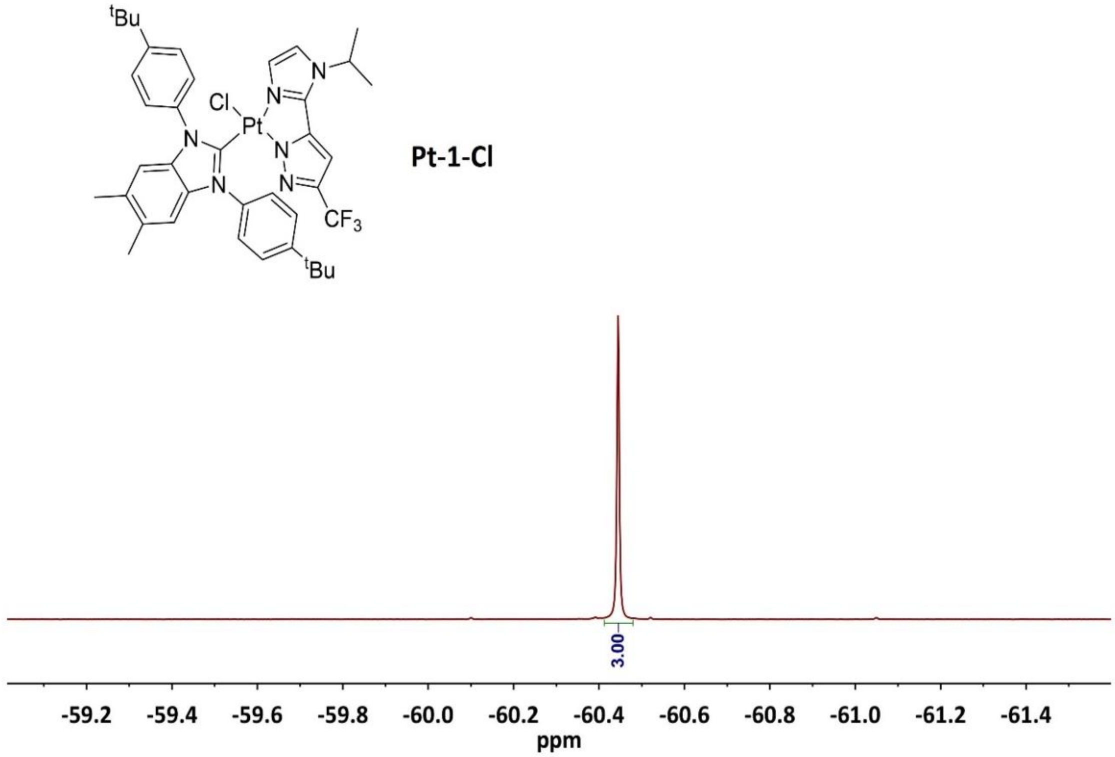

Figure S9. ^{19}F NMR spectrum (376 MHz) of **Pt-1-Cl** in $CDCl_3$ at 298 K.

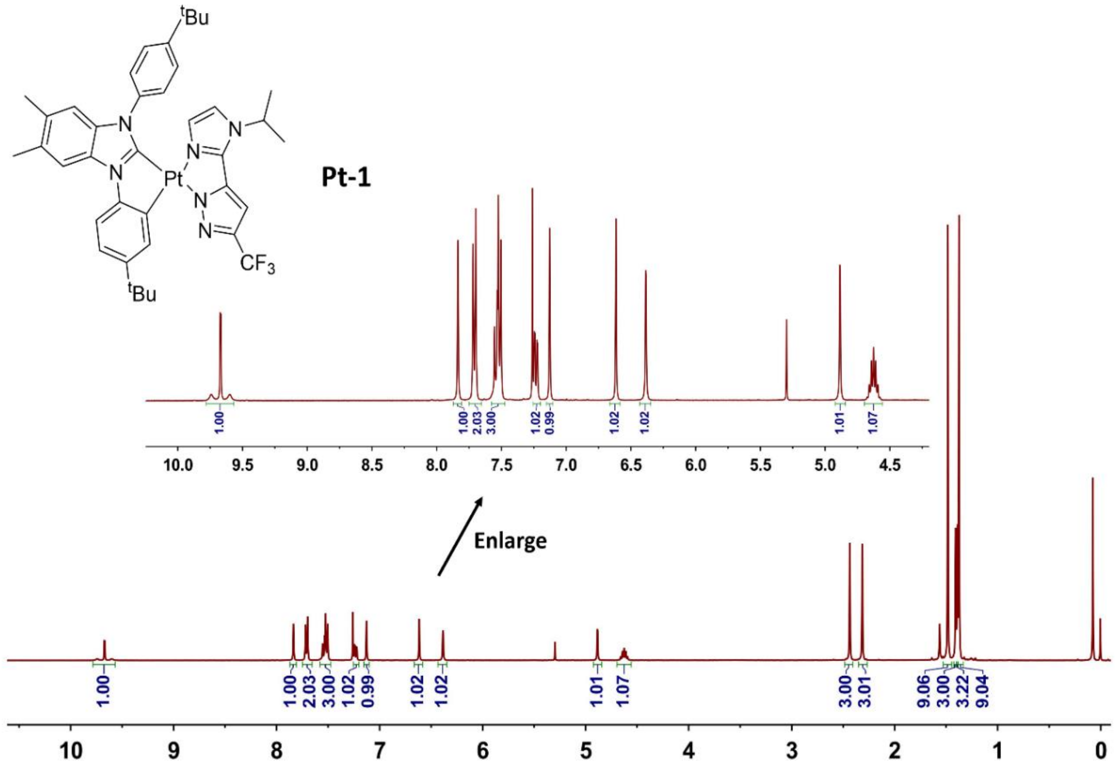

Figure S10. ^{1}H NMR spectrum (400 MHz) of **Pt-1** in $CDCl_3$ at 298 K.

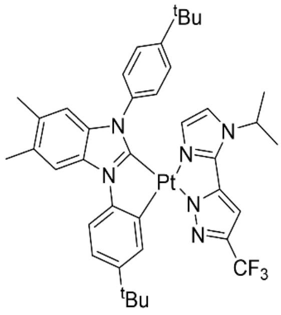

Pt-1

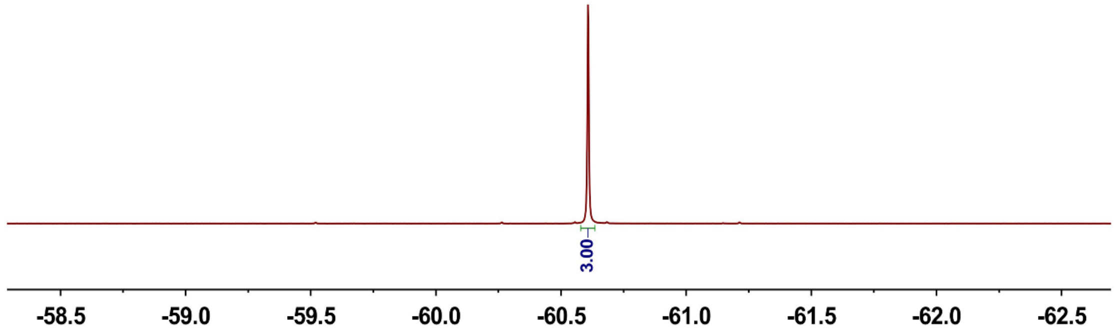

Figure S11. ^{19}F NMR spectrum (376 MHz) of **Pt-1** in $CDCl_3$ at 298 K.

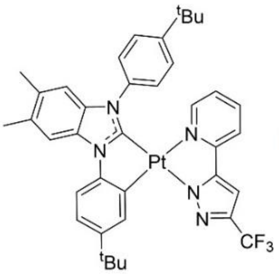

Pt-2

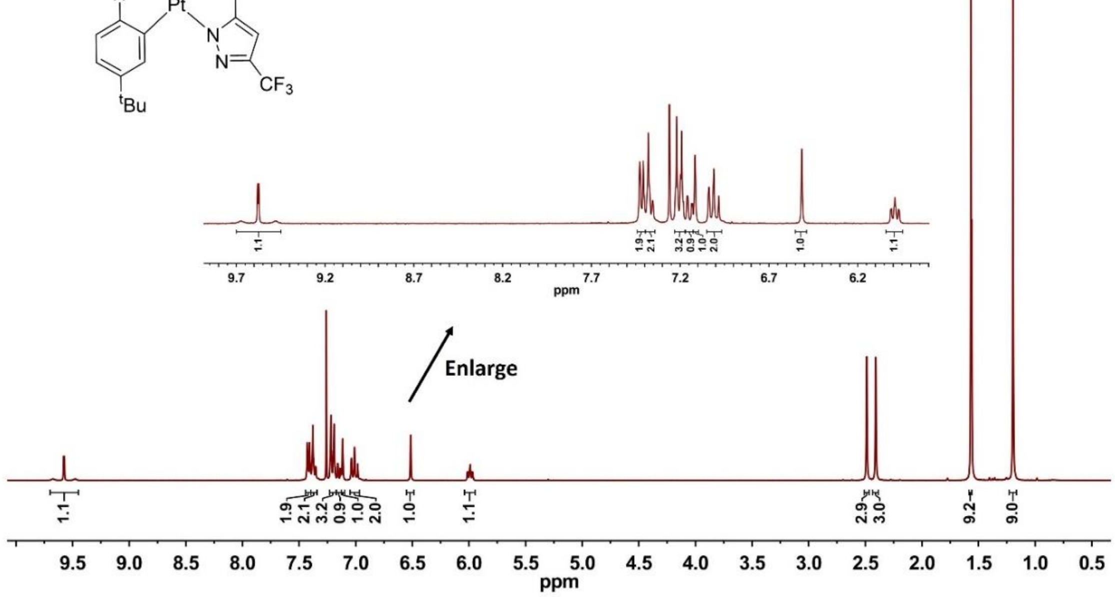

Figure S12. ^{1}H NMR spectrum (400 MHz) of **Pt-2** in $CDCl_3$ at 298 K.

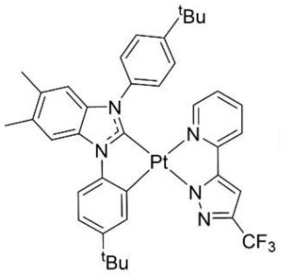

Pt-2

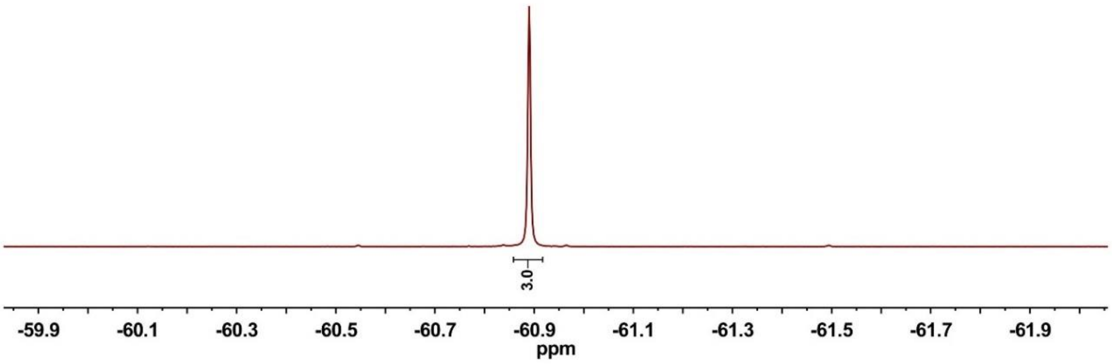

Figure S13. ^{19}F NMR spectrum (376 MHz) of **Pt-2** in $CDCl_3$ at 298 K.

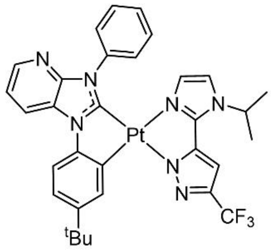

Pt-3a

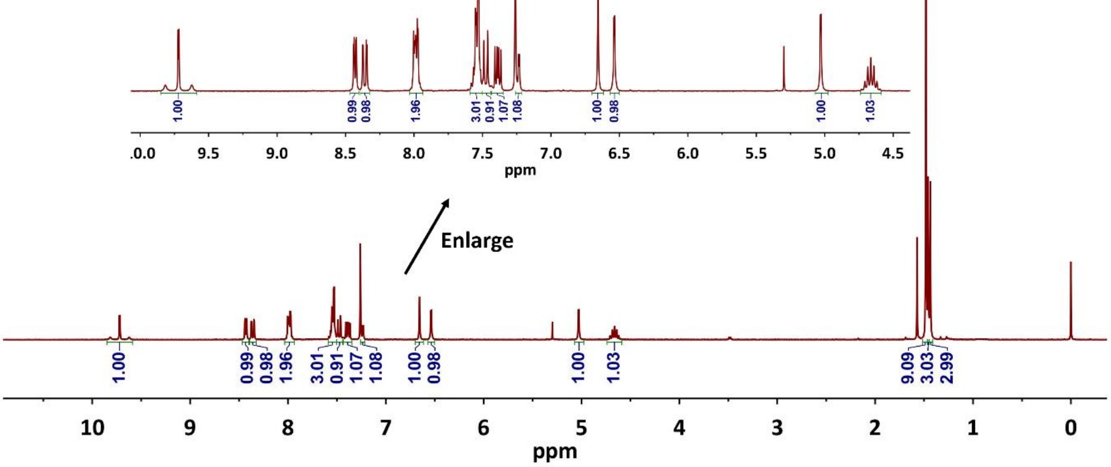

Figure S14. ^{1}H NMR spectrum (400 MHz) of **Pt-3a** in $CDCl_3$ at 298 K.

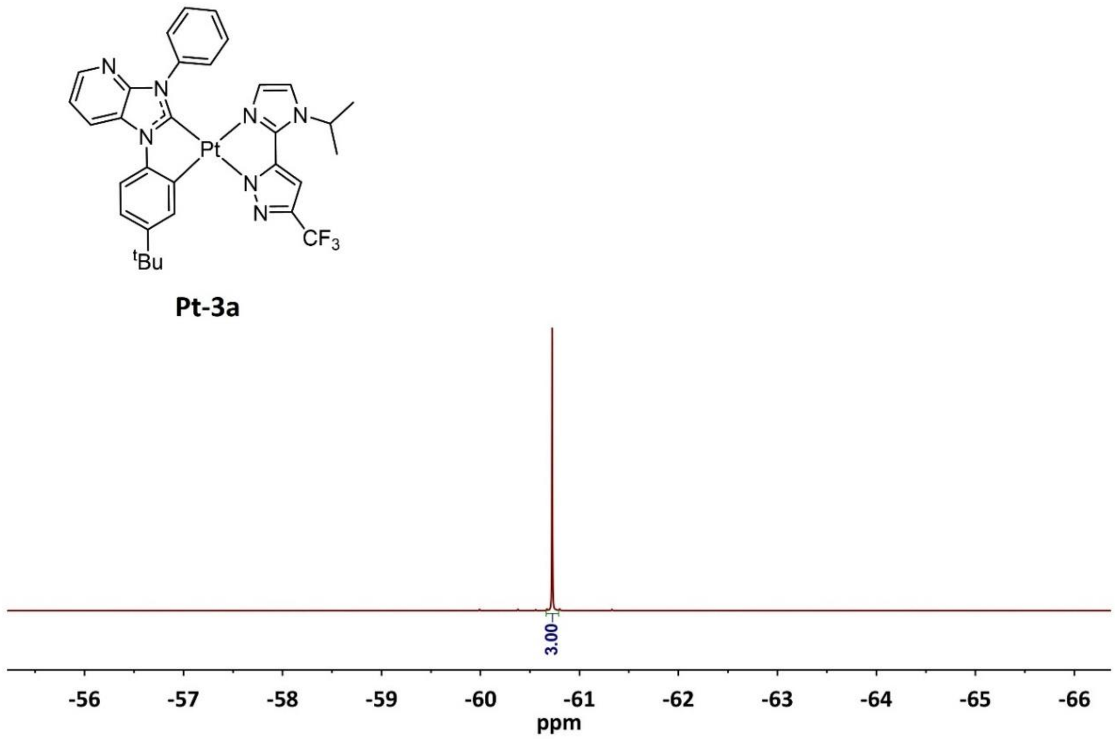

Figure S15. ^{19}F NMR spectrum (376 MHz) of **Pt-3a** in $CDCl_3$ at 298 K.

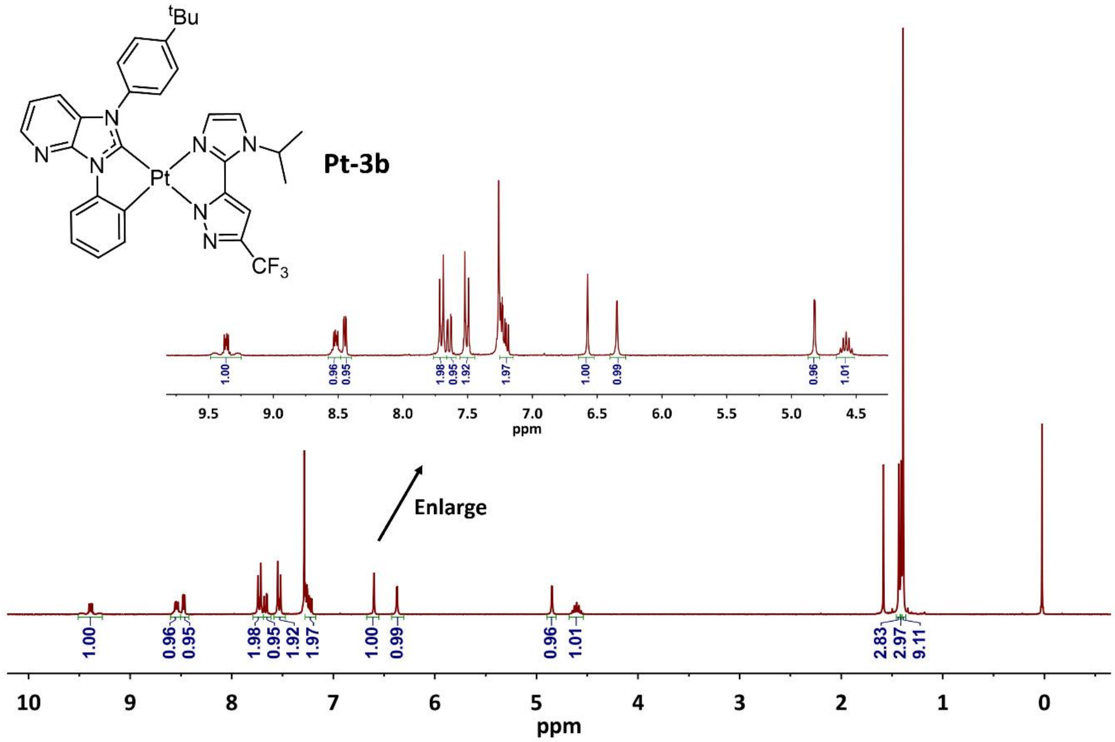

Figure S16. ^{1}H NMR spectrum (400 MHz) of **Pt-3b** in $CDCl_3$ at 298 K.

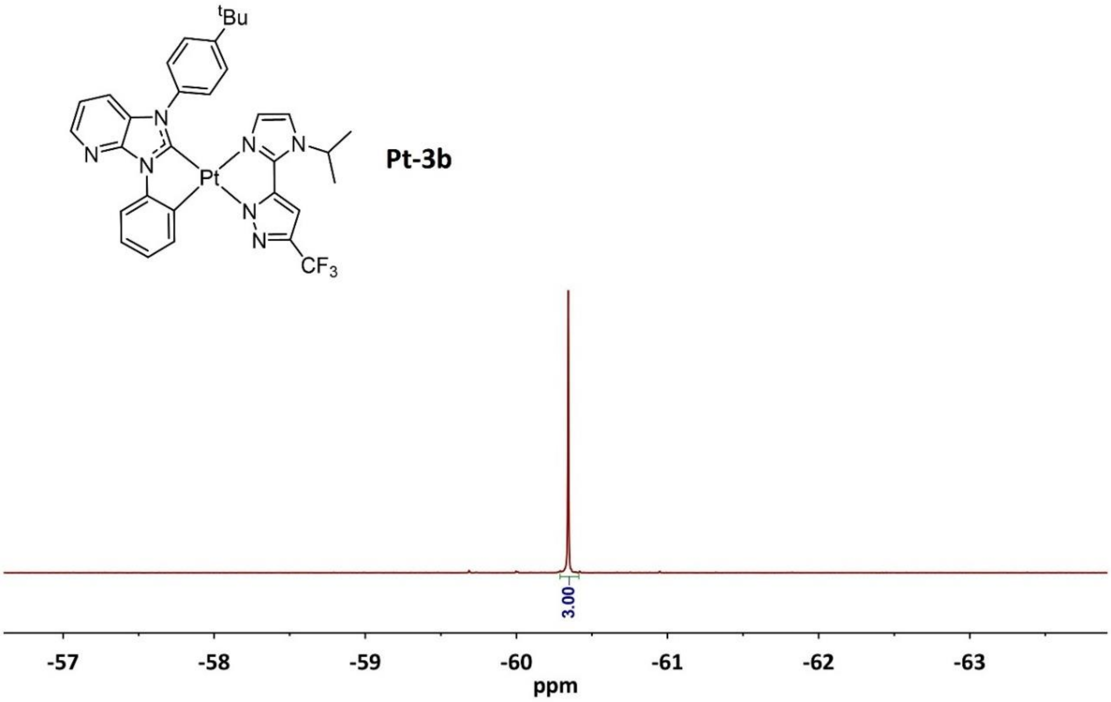

Figure S17. ^{19}F NMR spectrum (376 MHz) of **Pt-3b** in $CDCl_3$ at 298 K.

References

(1) Becke, A. D. Density-functional thermochemistry. III. The role of exact exchange. *J. Chem. Phys.* **1993,** *98* (7), 5648-5652.

(2) Weigend, F.; Ahlrichs, R. Balanced basis sets of split valence, triple zeta valence and quadruple zeta valence quality for H to Rn: Design and assessment of accuracy. *Phys. Chem. Chem. Phys.* **2005,** *7* (18), 3297-3305, DOI: 10.1039/B508541A.

(3) Weigend, F. Accurate Coulomb-fitting basis sets for H to Rn. *Phys. Chem. Chem. Phys.* **2006,** *8* (9), 1057-1065, DOI: 10.1039/B515623H.

(4) Grimme, S.; Ehrlich, S.; Goerigk, L. Effect of the damping function in dispersion corrected density functional theory. *J. Comput. Chem.* **2011,** *32* (7), 1456-1465, DOI: 10.1002/jcc.21759.

(5) Frisch, M. J.; Trucks, G. W.; Schlegel, H. B.; Scuseria, G. E.; Robb, M. A.; Cheeseman, J. R.; Scalmani, G.; Barone, V.; Mennucci, B.; Petersson, G. A.; Nakatsuji, H.; Caricato, M.; Li, X.; Hratchian, H. P.; Izmaylov, A. F.; Bloino, J.; Zheng, G.; Sonnenberg, J. L.; Hada, M.; Ehara, M.; Toyota, K.; Fukuda, R.; Hasegawa, J.; Ishida, M.; Nakajima, T.; Honda, Y.; Kitao, O.; Nakai, H.; Vreven, T.; Montgomery, J. A.; Peralta, J. E.; Ogliaro, F.; Bearpark, M.; Heyd, J. J.; Brothers, E.; Kudin, K. N.; Staroverov, V. N.; Kobayashi, R.; Normand, J.; Raghavachari, K.; Rendell, A.; Burant, J. C.; Iyengar, S. S.; Tomasi, J.; Cossi, M.; Rega, N.; Millam, J. M.; Klene, M.; Knox, J. E.; Cross, J. B.; Bakken, V.; Adamo, C.; Jaramillo, J.; Gomperts, R.; Stratmann, R. E.; Yazyev, O.; Austin, A. J.; Cammi, R.; Pomelli, C.; Ochterski, J. W.; Martin, R. L.; Morokuma, K.; Zakrzewski, V. G.; Voth, G. A.; Salvador, P.; Dannenberg, J. J.; Dapprich, S.; Daniels, A. D.; Farkas, Ö.; Foresman, J. B.; Ortiz, J. V.; Cioslowski, J.; Fox, D. J. Gaussian 16, Revision C.01. *Gaussian 16, Revision C.01; Gaussian Inc.* **2016**, Wallingford, CT.

(6) Miertuš, S.; Scrocco, E.; Tomasi, J. Electrostatic interaction of a solute with a continuum. A direct utilizaion of AB initio molecular potentials for the prevision of solvent effects. *Chem. Phys.* **1981,** *55* (1), 117-129.

(7) Miertuš̃, S.; Tomasi, J. Approximate evaluations of the electrostatic free energy and internal energy changes in solution processes. *Chem. Phys.* **1982,** *65* (2), 239-245.

(8) Adamo, C.; Jacquemin, D. The calculations of excited-state properties with Time-Dependent Density Functional Theory. *Chem. Soc. Rev.* **2013,** *42* (3), 845-856, DOI: 10.1039/C2CS35394F.

(9) Laurent, A. D.; Adamo, C.; Jacquemin, D. Dye chemistry with time-dependent density functional theory. *Phys. Chem. Chem. Phys.* **2014,** *16* (28), 14334-14356, DOI: 10.1039/C3CP55336A.

(10) Martin, R. L. Natural Transition Orbitals. *J. Chem. Phys.* **2003,** *118* (11), 4775-4777, DOI: 10.1063/1.1558471.

(11) Lu, T.; Chen, F. Multiwfn: A multifunctional wavefunction analyzer. *J. Comput. Chem.* **2012,** *33* (5), 580-592.

(12) Hirshfeld, F. L. Bonded-atom fragments for describing molecular charge densities. *Theo. Chim. Acta* **1977,** *44* (2), 129-138, DOI: 10.1007/BF00549096.

(13) de Souza, B.; Farias, G.; Neese, F.; Izsák, R. Predicting Phosphorescence Rates of Light Organic Molecules Using Time-Dependent Density Functional Theory and the Path Integral Approach to Dynamics. *J. Chem. Theory Comput.* **2019,** *15* (3), 1896-1904, DOI: 10.1021/acs.jctc.8b00841.

(14) Neese, F.; Wennmohs, F.; Becker, U.; Riplinger, C. The ORCA quantum chemistry program package. *J. Chem. Phys.* **2020,** *152* (22), 224108, DOI: 10.1063/5.0004608.

(15) Neese, F. Software update: The ORCA program system - Version 5.0. *WIREs Comput. Mol. Sci.*

2022, *12* (5), e1606, DOI: https://doi.org/10.1002/wcms.1606.
(16) van Lenthe, E.; Baerends, E. J.; Snijders, J. G. Relativistic regular two‐component Hamiltonians. *J. Chem. Phys.* **1993,** *99* (6), 4597-4610, DOI: 10.1063/1.466059.
(17) van Lenthe, E.; Baerends, E. J.; Snijders, J. G. Relativistic total energy using regular approximations. *J. Chem. Phys.* **1994,** *101* (11), 9783-9792, DOI: 10.1063/1.467943.
(18) Pye, C. C.; Ziegler, T. An implementation of the conductor-like screening model of solvation within the Amsterdam density functional package. *Theor. Chem. Acc.* **1999,** *101* (6), 396-408, DOI: 10.1007/s002140050457.
